# Identification of staphyloxanthin and derivates in yellow-pigmented *Staphylococcus capitis* subsp. *capitis*

**DOI:** 10.3389/fmicb.2023.1272734

**Published:** 2023-09-29

**Authors:** Katharina Siems, Katharina Runzheimer, Katarina Rebrosova, Lara Etzbach, Alina Auerhammer, Anna Rehm, Oliver Schwengers, Martin Šiler, Ota Samek, Filip Růžička, Ralf Moeller

**Affiliations:** ^1^Department of Radiation Biology, Institute of Aerospace Medicine, German Aerospace Center (DLR), Cologne, Germany; ^2^Department of Microbiology, Faculty of Medicine, Masaryk University and St. Anne’s University Hospital, Brno, Czechia; ^3^Institute of Nutritional and Food Sciences, Food Sciences, University of Bonn, Bonn, Germany; ^4^Department of Algorithmic Bioinformatics, Justus Liebig University Giessen, Giessen, Germany; ^5^Department of Bioinformatics and Systems Biology, Justus Liebig University Giessen, Giessen, Germany; ^6^Institute of Scientific Instruments of the Czech Academy of Sciences, Brno, Czechia

**Keywords:** *Staphylococcus capitis*, staphyloxanthin, coagulase-negative staphylococci (CoNS), carotenoids, bacterial pigments

## Abstract

**Introduction:**

*Staphylococcus capitis* naturally colonizes the human skin but as an opportunistic pathogen, it can also cause biofilm-associated infections and bloodstream infections in newborns. Previously, we found that two strains from the subspecies *S. capitis* subsp. *capitis* produce yellow carotenoids despite the initial species description, reporting this subspecies as non-pigmented. In *Staphylococcus aureus,* the golden pigment staphyloxanthin is an important virulence factor, protecting cells against reactive oxygen species and modulating membrane fluidity.

**Methods:**

In this study, we used two pigmented (DSM 111179 and DSM 113836) and two non-pigmented *S. capitis* subsp. *capitis* strains (DSM 20326^T^ and DSM 31028) to identify the pigment, determine conditions under which pigment-production occurs and investigate whether pigmented strains show increased resistance to ROS and temperature stress.

**Results:**

We found that the non-pigmented strains remained colorless regardless of the type of medium, whereas intensity of pigmentation in the two pigmented strains increased under low nutrient conditions and with longer incubation times. We were able to detect and identify staphyloxanthin and its derivates in the two pigmented strains but found that methanol cell extracts from all four strains showed ROS scavenging activity regardless of staphyloxanthin production. Increased survival to cold temperatures (−20°C) was detected in the two pigmented strains only after long-term storage compared to the non-pigmented strains.

**Conclusion:**

The identification of staphyloxanthin in *S. capitis* is of clinical relevance and could be used, in the same way as in *S. aureus*, as a possible target for anti-virulence drug design.

## 1. Introduction

*Staphylococcus capitis* (*S. capitis*) is a natural member of the human skin microbiome (Natsis and Cohen, 2018). However, it can also act as an opportunistic pathogen, causing bloodstream infections in neonates with many strains showing resistance to antibiotics (Decalonne et al., 2020; Wirth et al., 2020). Moreover, *S. capitis* has the ability to form resistant biofilms and is related to infections such as endocarditis, urinary tract infections, and catheter induced bacteremia (Cui et al., 2013; Cameron et al., 2015). *S. capitis* is divided into two subspecies: *S. capitis* subsp. *capitis* and *S. capitis* subsp. *ureolyticus* that show differences in urease activity, aerobic acid production from maltose, fatty acid profiles, and colony morphology (Bannerman and Kloos, 1991). Because *S. capitis* is naturally part of the human microbiome, it is often found in crewed spaceflight environments, such as the International Space Station (ISS), which is generally dominated by human-associated microorganisms (Mora et al., 2019; Sobisch et al., 2019). The current literature on spaceflight-induced effects on bacterial physiology is inconclusive, therefore, we previously performed a comprehensive comparison between an *S. capitis* subsp. *capitis* strain isolated from the ISS to other *S. capitis* subsp. *capitis* strains isolated on Earth (Siems et al., 2022). Within that study, no significant differences were detected in the spaceflight isolated strain in regards to antibiotic resistance, radiation tolerance or virulence genes. However, it was found that the ISS-isolated strain (DSM 111179) showed delayed yellow colony pigmentation which was only present in one other strain (DSM 113836) but absent in the other strains. This other yellow pigmented strain was isolated from a forehead skin swab from one of the subjects in a bedrest study, where the subjects were exposed to a 6° head down tilt for 60 days in order to simulate fluidic shifts similar to the shift observed in astronauts during spaceflight. Based on the results of our previous study, we assume that the pigments are carotenoids, more specifically xantophylls, since they show the typical absorption spectrum with absorption maxima between 400 and 500 nm (Marshall and Wilmoth, 1981). Furthermore, we found that these two pigmented strains showed an increased resistance to hydrogen peroxide, which is of great concern in the context of spaceflight, as surfaces in the ISS are frequently cleaned and disinfected by astronauts with wipes containing H_2_O_2_ (ViroxTechnologies, 2018). In other staphylococci, production of carotenoid pigments serves important biological functions. In *Staphylococcus aureus* (*S. aureus*), the golden pigment staphyloxanthin is an important virulence factor. It protects cells from reactive oxygen species (ROS), released by immune cells upon phagocytosis (Liu et al., 2005; Clauditz et al., 2006) and stabilizes the cell membrane by decreasing membrane fluidity (Mishra et al., 2011). Moreover, staphyloxanthin was found to have protective function in *S. aureus* against UV (254 nm) (Pannu et al., 2019).

Interestingly, the biosynthetic gene cluster of staphyloxanthin has been detected before throughout the *Staphylococcus* genus (Salamzade et al., 2023), but the compound staphyloxanthin has not been extracted and identified in species other than *S. aureus* and *Staphylococcus xylosus* (*S. xylosus*) (Seel et al., 2020). In the first description of *S. capitis* subsp. *capitis,* this subspecies was described as unpigmented (Bannerman and Kloos, 1991) but in our previous study we detected yellow pigmentation in two strains that were identified as *S. capitis* subsp. *capitis.* Thus, the aim of this study was to (i) determine the conditions under which colony pigmentation occurs in *S. capitis* subsp. *capitis* strains, (ii) identify the produced pigments and (iii) investigate whether pigmentation leads to advantages in survival during ROS and temperature stress.

## 2. Materials and methods

### 2.1. Strains

In this study, we used four different strains that were previously identified as *S. capitis* subsp. *capitis* (Siems et al., 2022). The type strain, DSM 20326^T^ obtained from the German Collection of Microorganisms and Cell Cultures (DSMZ), referred to in this study as D2^T^ was used as a reference. Additionally, we tested the strains DSM 31028 (D3), DSM 111179 (K1) and DSM 113836 (H17). Strain D3 was isolated inside a cleanroom of a spacecraft assembly facility, strain K1 was isolated within an exposure experiment performed inside the International Space Station (ISS) (Sobisch et al., 2019) and strain H17 was isolated from the forehead of a human subject during an artificial gravity bedrest study (Siems et al., 2022).

### 2.2. Cultivation

The strains were cultivated on plates that were incubated at 37°C for 24 h up to 48 h and further stored at room temperature ~21°C (RT). In this study we used tryptic soy agar (TSA, 17 g/L casein peptone, 3 g/L soy peptone, 5 g/L NaCl, 2.5 g/L K_2_HPO_4_, 2.5 g/L glucose, 15 g/L agar, pH 7.3), Reasoners 2A agar (R2A, 0.5 g/L yeast extract, 0.5 g/L proteose peptone, 0.5 g/L casamino acids, 0.5 g/L dextrose, 0.5 g/L soluble starch, 0.3 g/L Na-pyruvate, 0.3 g/L K_2_HPO_4_, 0.05 g/L MgSO_4_, 15 g/L agar, pH 7.2) and Columbia blood agar (CBA, 23 g/L peptone, 1 g/L starch, 5 g/L NaCl, 50 mL/L sheep blood, 14 g/L agar, pH 7.5).

### 2.3. Raman spectroscopy of colonies on agar

To acquire Raman spectra, the strains were cultivated on CBA, TSA, and R2A agar plates at 37°C for 24 h, 48 h, and 144 h (48 h at 37°C, 4 days RT). Following cultivation, we acquired Raman spectra from bacterial colonies with the commercial Renishaw Raman spectrometer (Renishaw inVia Raman Spectrometer, Renishaw plc., Wotton-under-Edge, United Kingdom) using a 785 nm single-mode diode laser as an excitation source. The laser beam was focused onto a single colony by a microscope objective (Leica, Wetzlar, Germany: N PLAN EPI, magnification 50×, numerical aperture 0.75, working distance 0.5 mm) with a laser spot dimensions of approx. 2 μm × 10 μm, and a full axial depth of the excitation region at 8 μm (Renishaw, 2003; Mlynarikova et al., 2015). Since the laser beam was focused onto the surface of the colony, signal contribution from the underlying medium was suppressed (Mlynarikova et al., 2015). Individual spectral acquisitions in the range 614–1,724 cm^−1^ took 15 s. Before each spectral acquisition, the laser beam refocussed onto the colony surface to stay within the focal depth of the laser beam excitation and the imaging optics. To account for possible variability of the cells forming the colony and variable colony height, spectra from different colony parts were taken. In total, ten measurements per strain were obtained from at least three colonies each. Acquired Raman spectra was analyzed using a standard multivariate principle component in-house program using the MATLAB software (MathWorks, Natick, MA, United States). First, the spectra were treated with rolling circle filtering (2 passes, 700 points circle radius) to suppress any background fluorescence. Subsequently, high-frequency noise was removed using Savitzky–Golay filtering (2nd order, width 7 points). The spectra were normalized to a peak assigned to phenylalanine (approx. 1,004 cm^−1^) (Bernatova et al., 2013; Mlynarikova et al., 2015). Comparison of spectra of individual strains was made using principal component analysis (PCA). Groups representing the individual strains were marked by ellipsoids with a Mahalanobis distance of 2.15, corresponding to a 90% confidence (De Maesschalck et al., 2000; Rebrošová et al., 2017) interval.

### 2.4. Pigment extraction and HPLC-DAD analysis

For pigment extraction, R2A agar plates were inoculated with 100 μL of cell suspensions each from overnight cultures in R2A liquid media. Plates were incubated for 24 h at 37°C and stored at RT for an additional 3 days. Biomass from five agar plates was collected with sterile inoculation loops and transferred into tubes containing 1 mL H_2_O. Suspensions were centrifuged for 10 min at 14000 × *g*. Supernatants were discarded and the pellets frozen at −20°C. For further analysis, pellets were thawed and 0.3 g biomass was transferred to a Lysing Matrix B tube (MP Biomedicals, Irvine, CA, United States) with 1.5 mL methanol (≥99.9%) for each pellet. Samples were heated at 55°C for 5 min and treated three times for 40 s in a high-speed benchtop homogenizer (FastPrep, MP Biomedicals) at 6 m/s. Afterwards, tubes were centrifuged at 14,000 × *g* for 10 min and the supernatant transferred to HPLC vials. Methanol was evaporated (Reacti-Vap/Therm, Thermo Fisher Scientific Inc., Waltham, MA, United States) and dried samples were stored at −80 °C. Prior to analysis, samples were dissolved in 50:50 MTBE/MeOH with 0.1% BHT and filtered through 0.2 μm syringe filters. HPLC-DAD analysis was performed on a Prominence UFLC system (Shimadzu, Kyoto, Japan) equipped with two Nexera X2 LC-30AD high-pressure gradient pumps, a Prominence DGU-20A5R degasser, a Nexera SIL-30AC Prominence autosampler (15°C, injection volume 10 μL), a CTO-20AC Prominence column oven 27°C, and a SPD-M20A Prominence diode array detector. The carotenoids were detected at a wavelength of 450 nm. An Accucore^™^ C30 column (Thermo Fisher Scientific, Braunschweig, Germany) with a length of 150 mm, a diameter of 2.1 mm and a particle size of 2.6 μm was selected for the separation of the carotenoids. The two eluents A and B of methanol/MTBE/H_2_O (85/5/10, v/v/v) and (11/85/4, v/v/v) (methanol: MS grade, MTBE: ≥99.0%), respectively, were used. β-carotene (purity >99%, Extrasynthese, Lyon, France) was used as an external standard. The gradient program was the following: 0 min, 0% B; 2 min, 5% B; 9 min, 30% B; 24 min, 30% B; 26 min, 70% B; 28 min, 70% B; 29 min, 100% B; 31 min, 100% B; 32 min, 0% B; 35 min, 0% B with a flow rate of 0.4 mL/min. Data acquisition and processing were performed by the LabSolutions software version 5.85 (Shimadzu, Kyoto, Japan).

### 2.5. Identification of pigments by HPLC-DAD-APCI-MS^n^

For tentative identification of the pigments from strain K1 and H17, we performed HPLC-DAD-APCI-MS^n^ (HPLC-DAD: high performance liquid chromatography-diode array detection, APCI: atmospheric pressure chemical ionization, MS: mass spectrometry). The biomass of 5 days old inoculated 2× R2A plates of the pigmented strains K1 and H17, and the biomass of D2^T^ (for reference) was collected as described before but here, carotenoid extraction was performed according to Kaiser et al. (2007) and Seel et al. (2020). HPLC-DAD-APCI-MS^n^ was performed as described in Etzbach et al. (2018). The analysis for the identification was performed on an Acquity UPLC I-Class system (Waters, Milford, MA, United States), consisting of a binary solvent manager, a sample manager-FL (15°C, injection volume 10 μL), a column oven at 27°C and a PDA eλ detector. The UPLC was coupled to an LTQ-XL ion trap mass spectrometer with an APCI ionization source (Thermo Fisher Scientific Inc., Waltham, MA, United States) operating in positive ion mode in a mass range of *m*/*z* 200–1,300. The chromatographic conditions (column, gradient program, flow rate, injection volume) were the same as mentioned above. Both eluents A and B were spiked with 5 mmol/L ammonium formate to improve ionization. Further parameters and settings for the mass spectrometry are summarized in Supplementary Table S1. Interpretation of the chromatograms was performed based on data from Bull (1970), Breithaupt et al. (2001), Pelz et al. (2005) and Kim et al. (2016).

### 2.6. DPPH radical scavenging assay

2,2-diphenyl-1-picrylhydrazyl (DPPH) was used to determine the radical scavenging activity of the methanol extracts according to Sendra et al. (2006) with modification from Yao and Qi (2016). In a 96-well-plate, 175 μL of DPPH solution (0.1 mmol/L) was incubated with 25 μL of methanol extracts of the four strains. 175 μL methanol with 25 μL of extracts were used as blanks. H_2_O was used as negative control and ascorbic acid (5 mg/mL) as positive control. Absorbance was measured at RT every 10 min at a wavelength of 517 nm over a time span of 120 min in total using a microplate reader (Infinite M200 PRO, Tecan, Männedorf, Switzerland). The percentage of scavenged DPPH was calculated according to Moore and Yu (2007) with the following formula:


$$\%\;\mathrm{DPPH}\;\mathrm{scavenged}=\left(1-\frac{A_{\mathrm{extract}}-A_{\mathrm{blank}}}{A_{H2O}-A_{\mathrm{blank}}}\right)\times 100$$

### 2.7. Genomic analysis

Genomes of the four *S. capitis* subsp. *capitis* strains have been sequenced and assembled previously (Genbank, BioProject: PRJNA849394) (Siems et al., 2022). For this study, the assembled genome sequences were annotated with Bakta (v1.7.0) (Schwengers et al., 2021) using the full database version 5.0. The *crt* operon, involved in the synthesis of staphyloxanthin, was aligned and visualized using clinker (v0.0.27) (Gilchrist and Chooi, 2021). The alignment of the amino acid sequences for each gene were performed using EDGAR (Dieckmann et al., 2021) and MUSCLE (v3.8) (Edgar, 2004). Alignment of nucleic acid sequences of *crtN* was performed with CLUSTAL O (1.2.4) (Madeira et al., 2022).

### 2.8. Temperature experiments

Since carotenoids modulate membrane fluidity of staphylococci (Seel et al., 2020) and therefore might influence survival under different temperatures, we tested the survival of the four *S. capitis* subsp. *capitis* strains at RT (~20°C), 4°C, −20°C as well as 37°C, 50°C and 60°C. For this, 50 mL R2A was inoculated and incubated at 37°C for 18 h shaking at 140 rpm. Cells were harvested by centrifugation at 14,000 × *g* for 10 min and washed with phosphate buffered saline (PBS, 7.0 g/L Na_2_HPO_4_, 3.0 g/L KH_2_PO_4_, 4.0 g/L NaCl, pH 7.5). Cell suspensions were aliquoted with a volume of 1 mL into 1.5 mL tubes. The number of CFU/mL was determined by standard plate count using R2A agar plates. For testing cold temperatures, tubes were stored at RT, 4°C, and − 20°C. After 24 h, CFU/mL was determined and the tubes containing the cell suspensions were stored again at the according temperatures. After 44 days, CFU/mL was determined again. For testing high temperatures, cell suspensions were incubated for 1 h at 37°C, 50°C and 60°C before CFU/mL was determined. Statistical significance was tested by performing one-way analysis of variance (ANOVA) for each time point followed by Holm-Sidak method for pairwise multiple comparisons, where *p* < 0.05 was considered significant.

## 3. Results

### 3.1. Colony morphology on different media

**Figure 1:**
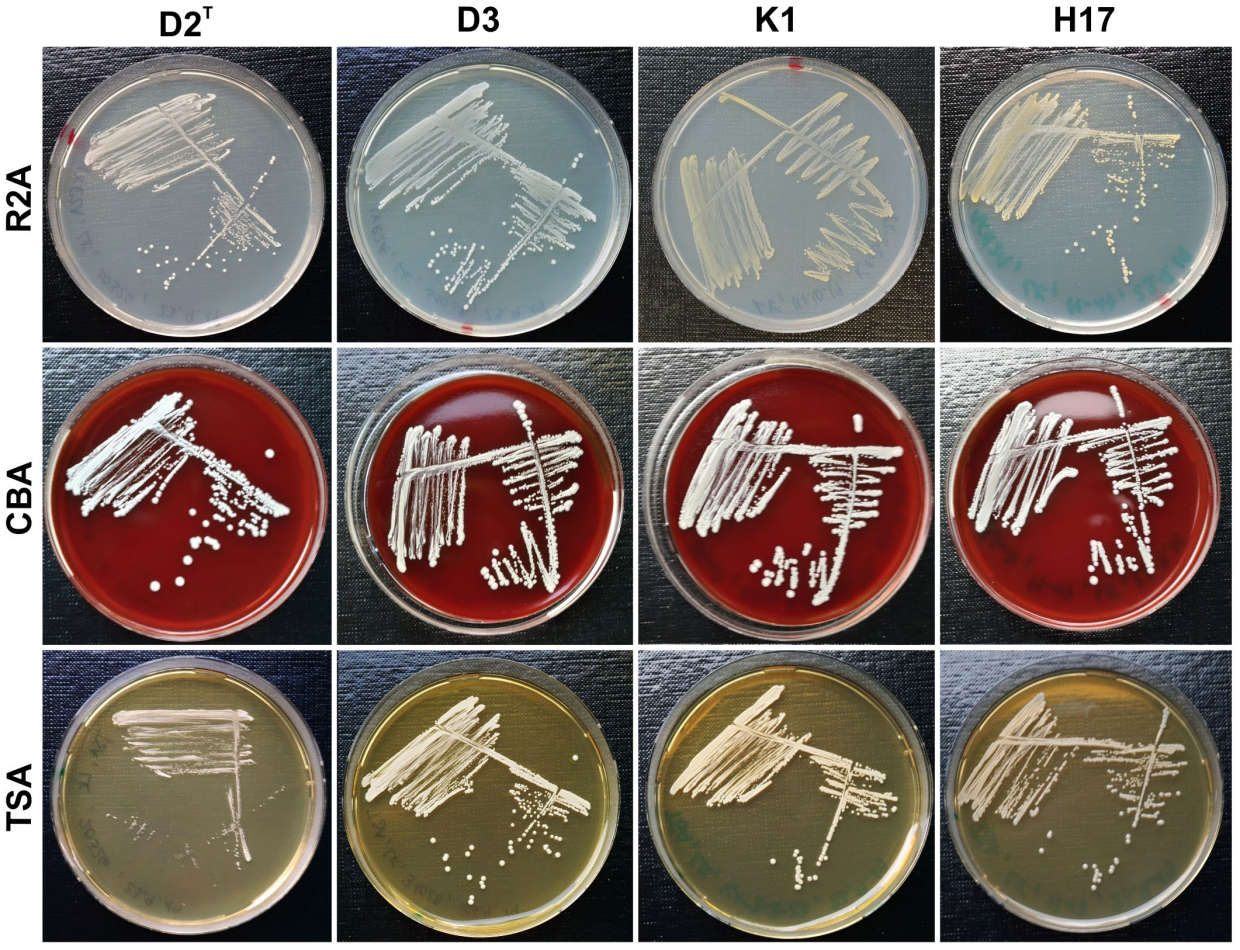
Colony morphology of strains D2^T^, D3, K1 and H17 cultivated on R2A, CBA and TSA plates (⌀ 9 cm) for 48 h at 37°C.

Figure 1 shows the colony morphology of the four different *S. capitis* subsp. *capitis* strains on Reasoners 2A agar (R2A), Columbia blood agar (CBA) and tryptic soy agar (TSA) after incubation for 48 h at 37°C. The colony morphology after 24 h of incubation at 37°C is shown in Supplementary Figure S1 and after 72 h (48 h 37°C + 24 h RT) in Supplementary Figure S2. Macroscopic inspection of colonies revealed increased growth of all four strains on CBA compared to the other two types of media. On CBA, the strains showed a similar morphology with no difference in colony shape, size or coloration at all time points. The colonies appeared round and white with no hemolysis. On TSA and R2A, strain D2^T^ showed reduced growth compared to the other three strains at all timepoints. Generally, colony size in all strains was correlated with decreasing amounts of nutrients available in the media (R2A < TSA < CBA). Strains K1 and H17 exhibited yellow colony pigmentation on R2A and TSA plates. This yellow pigmentation seemed to increase over time and was visually detectable on R2A agar already after 24 h and on TSA after 72 h of incubation.

### 3.2. Raman spectroscopy of colonies on agar

**Figure 2:**
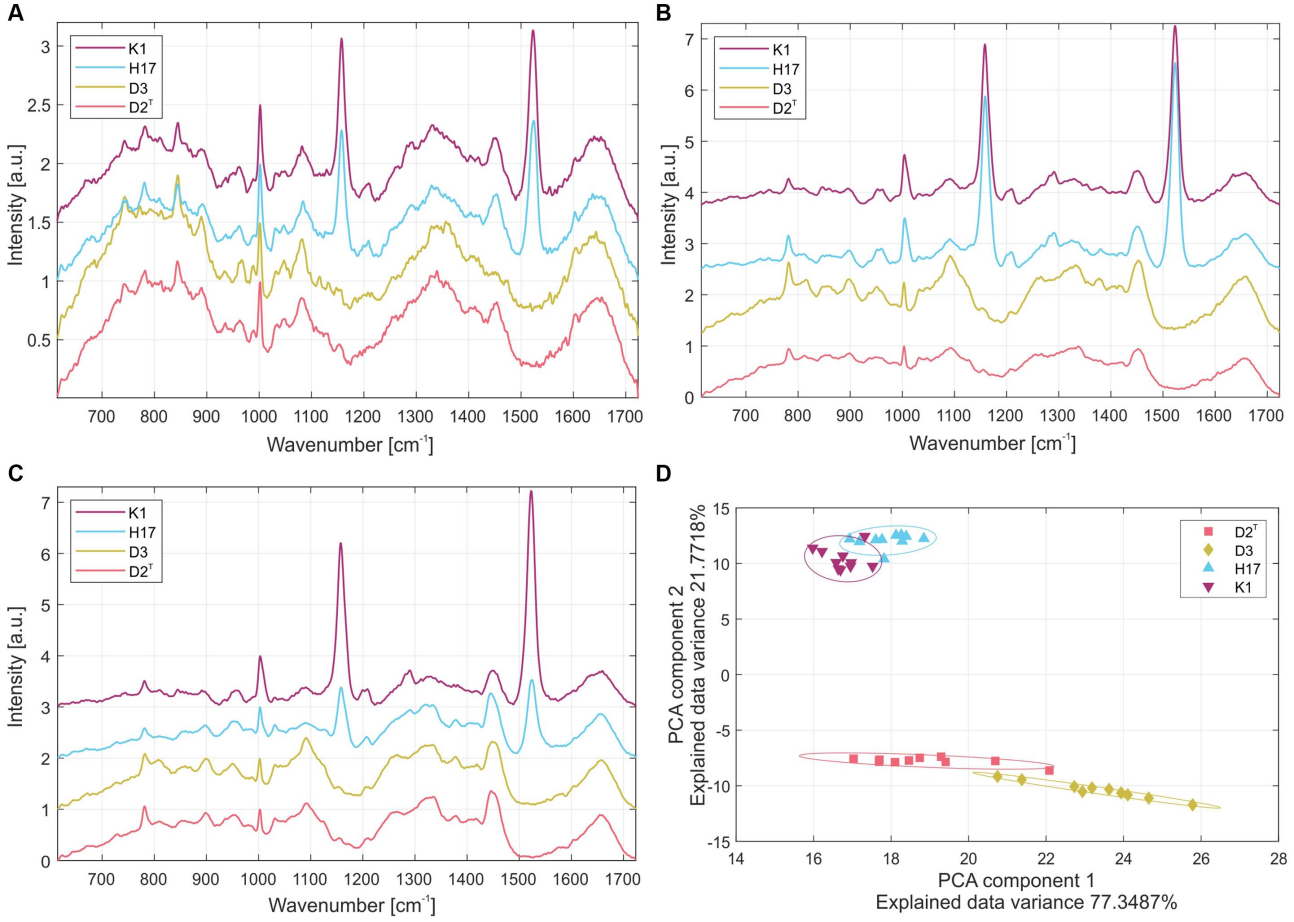
Normalized average Raman spectra from colonies of the four *S. capitis* subsp. *capitis* strains K1 (violet), H17 (blue), D3 (yellow) and D2^T^ (red) after incubation on R2A agar at 37°C for **(A)** 24 h, **(B)** 48 h and **(C)** 48 h with additional 4 days storage at RT. **(D)** Principal component analysis of Raman spectroscopy results of colonies grown on R2A at 37°C for 48 h. Individual strains were marked by ellipsoids with a Mahalanobis distance of 2.15 corresponding to a 90% confidence interval.

**Figure 3:**
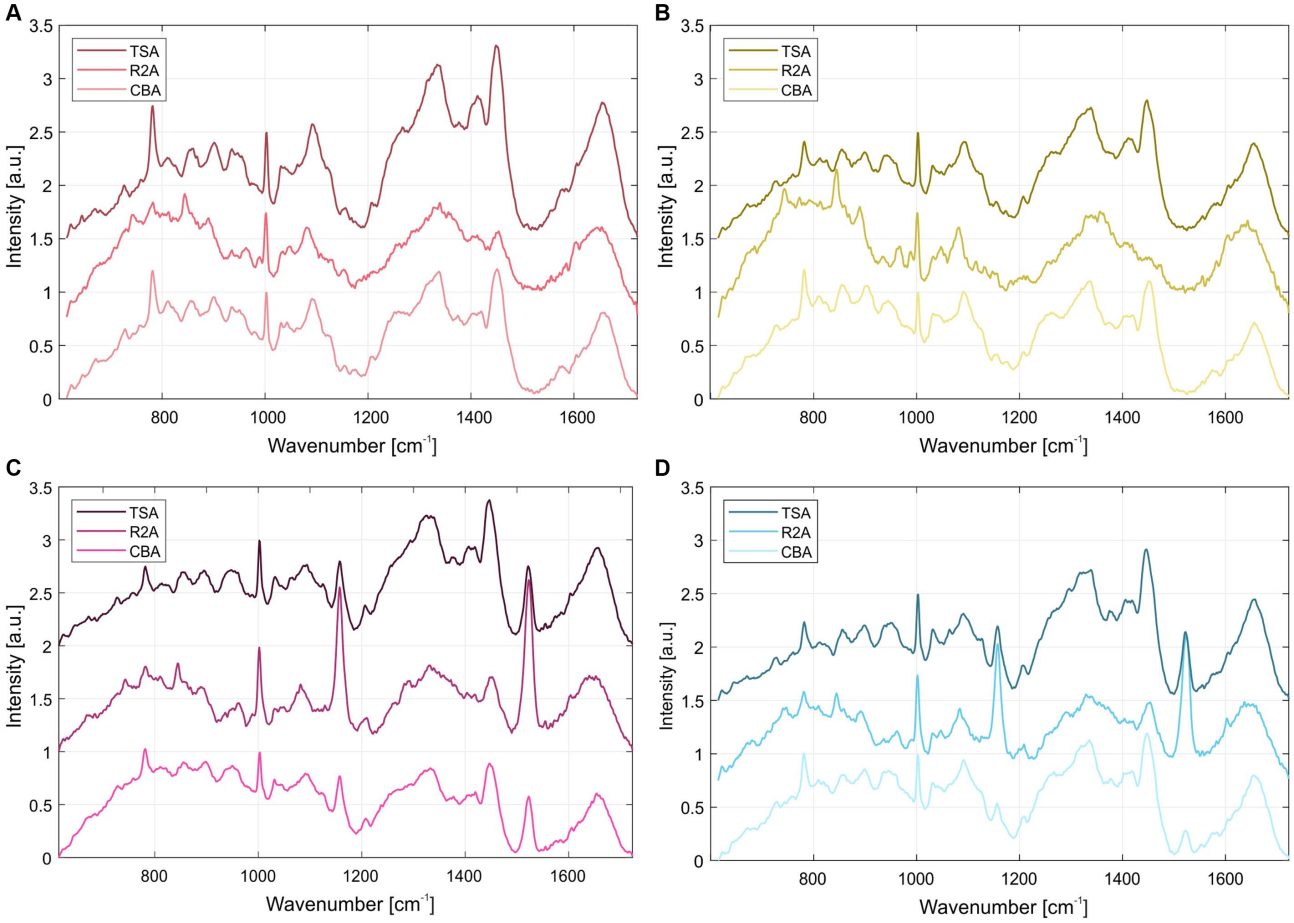
Normalized average Raman spectra of *S. capitis* subsp. *capitis* strains after 24 h incubation at 37°C on different media for strains **(A)** D2^T^, **(B)** D3, **(C)** K1 and **(D)** H17. From top to bottom spectra for colonies grown on TSA, R2A, and CBA are shown.

The normalized average Raman spectra for the colonies of the four *S. capitis* subsp. *capitis* strains on R2A agar are displayed in Figure 2. Colonies were measured after incubation times of 24 h (Figure 2A) and 48 h (Figure 2B) at 37°C as well as, 48 h at 37°C and 4 days storage at RT (Figure 2C). Supplementary Table S2 lists the suggested assignments of the peaks in the spectra to bacterial cell components. For all incubation times, the Raman spectra of K1 and H17 show two distinct peaks at 1160 cm^−1^ and 1,525 cm^−1^. These peaks are missing or are only weakly expressed in D2^T^ and D3. These peaks correspond to =C–C= and –C=C-bonds of carotenoid pigments, respectively (Verma et al., 1984). Moreover, according to the loadings, the most striking changes corresponded to the carotenoid peaks (Supplementary Figure S3). Principal component analysis (PCA) confirmed the differences between the pigmented and non-pigmented strains, as can be seen in Figure 2D with the two groups forming distinct clusters, K1 with H17, and D2^T^ with D3. The intensity of carotenoid peaks increased for both pigmented strains from 24 h to 48 h. This was also confirmed by visually observing the change in color of the colonies from cream with a yellowish tinge to yellow. For K1, the intensity of the peaks remained on a similar level after additional storage for 4 days at RT (Figure 2C). Whereas in H17, the intensity of the peaks decreased (Figure 2C). However, strain H17 generally showed pigment-associated variances within Raman spectra (Supplementary Figure S4). Following the direct comparison of the Raman spectra of colonies of the same strain on different media, it was shown that D2^T^ (Figure 3A) and D3 (Figure 3B) colonies did not exhibit the characteristic carotenoid peaks on any of the tested media. In contrast, strain K1 (Figure 3C) and H17 (Figure 3D) showed carotenoid-assigned peaks in the normalized average Raman spectra on all tested media. Intensity of the peaks seemed to be the highest in colonies grown on R2A compared to colonies grown on CBA and TSA (for reference, Supplementary Figure S5 shows the Raman spectra of the different media without bacterial colonies). The same trend was observed in H17 (Figure 3D) where the lowest intensity was observed on CBA.

### 3.3. Detection and identification of pigments

**Figure 4:**
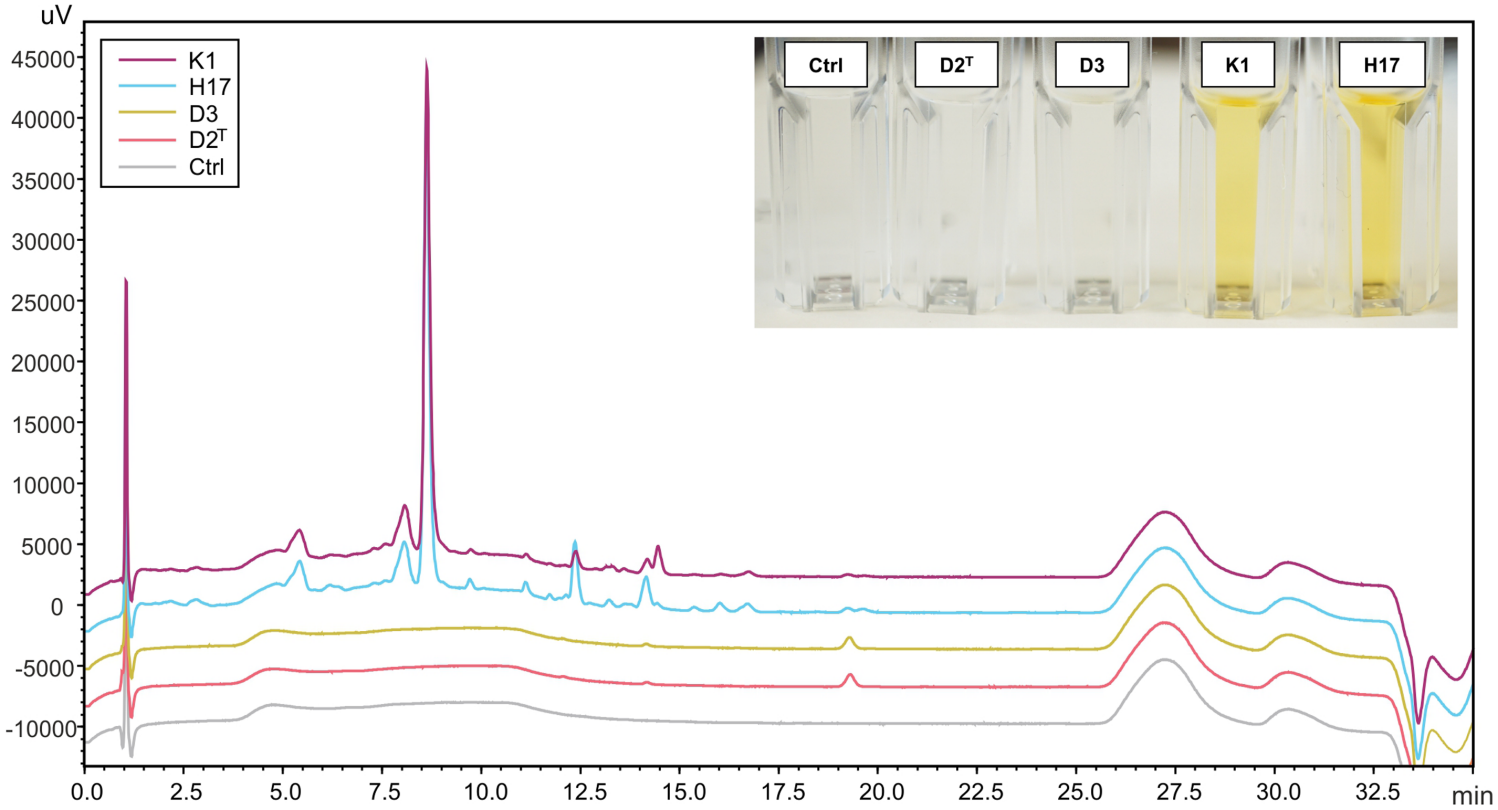
HPLC-DAD chromatogram (450 nm) of methanol extracts from *S. capitis* subsp. *capitis* strains K1 (violet), H17 (blue), D3 (yellow), D2^T^ (red) grown on R2A agar for 24 h at 37°C and methanol control (Ctrl). An image of the methanol extracts in cuvettes is shown in the top right corner. Cuvettes are labelled according to strains.

After mechanical lysis of all four strains, only the methanol extracts of strains K1 and H17 were colored yellow (photographic image in Figure 4). For further detection of carotenoids, HPLC-DAD analysis was performed on the methanol extracts from all four strains. Figure 4 shows the corresponding chromatogram at 450 nm. Only one peak was detected in the extracts of the unpigmented strains D2^T^ and D3 at a retention time of 19.5 min. Whereas, several peaks were detected in the two yellow pigmented strains K1 and H17 that indicated the presence of several carotenoid compounds.

For identification of carotenoids in the pigmented methanol extracts from strains K1 and H17, an additional HPLC-DAD system with a coupled mass spectrometer (HPLC-DAD-APCI-MS^n^) was used. D2^T^ was included in this analysis as a non-pigmented reference. The chromatograms of K1 and H17 showed seven major peaks which also exhibited absorptions in wavelengths typical for carotenoids (400 to 500 nm, Supplementary Figure S6). The UV-spectra of peak 1–6 are shown in Supplementary Figure S7, whereby the UV-spectra of peak 7 was low in resolution due to low compound concentration. D2^T^ only showed one minor peak with an indistinct UV-spectrum at a retention time (*t*_r_) of 15.59 min (Supplementary Figure S8), which corresponds to peak seven in the spectra of K1 and H17. The tentative identification of the detected carotenoids is shown in Table 1, where %III/II was calculated as the ratio of the height of the longest wavelength absorption peak (III) and that of the middle absorption peak (II) with the minimum between the two peaks as baseline, expressed in %. The first eluting carotenoid was tentatively identified as all-*trans* 4,4′-diaponeurosporenoic acid. The according mass spectra and structure are shown in Supplementary Figure S9. The second eluting carotenoid was tentatively identified as all-*trans*-staphyloxanthin and subsequent compounds 3–6 were likely to represent staphyloxanthin-like carotenoids. The according mass spectra and structure of staphyloxanthin are shown in Supplementary Figure S10. The pigment eluted last was 4,4′-diaponeurosporene, which was detected in small amounts in all samples. Considering the relative distribution of carotenoid proportions, the first eluate all-*trans*-4,4′-diaponeurosporenoic acid, a staphyloxanthin precursor, was the most abundant in K1 and H17 with 37% and 31%, respectively. This was followed by all-*trans*-staphyloxanthin with 26%–30%, and the four different staphyloxanthin-like compounds with a highly variable proportion ranging from 3%–18%. The last eluted pigment 4,4′-diaponeurosporene was the least represented in K1 and H17, while in D2^T^ it represented the only detectable compound.

**Table 1:** Tentatively identified carotenoids and the corresponding retention time (*t*_R_) in minutes, the absorption maxima (*λ*_max_), shape of absorption spectrum as percent value (% III/II), detected mass spectrum [(M + H)^+^], MS^2^ (*m*/*z*) and MS^3^ (*m*/*z*) of carotenoid extract from K1 (sh, shoulder in absorptions spectrum; nd, not detected) and relative distribution (in %) of the carotenoid fractions 1 to 7 of K1 and H17.

	Tentatively identified carotenoid	*t*_R_ (min) UV	*λ*_max_ (nm)			(% III/II)	(M + H)^+^ (*m*/*z*)	MS^2^ (*m*/*z*)	MS^3^ (*m*/*z*)	Relative distribution (%)	
										K1	H17
1	All-*trans*-4,4′-diaponeurosporenoic acid	4.46	428sh	452	478	51.51	433	[433]: 415, 387, 377, 334, 309, 291, 231, 209, 159	433-->415: 397, 387, 359, 345, 277, 237	37.35	31.26
2	All-*trans*-staphyloxanthin	10.02		466	489sh	nd	819	433		26.19	30.18
3	Staphyloxanthin like	11.31		466	489sh	nd	847	433		18.41	17.59
4	Staphyloxanthin like	12.57		466	489sh	nd	861	433		2.84	4.27
5	Staphyloxanthin like	13.10		466	489sh	nd	875	433		9.34	9.18
6	Staphyloxanthin like	14.87		466	489sh	nd				3.94	5.61
7	4,4′-diaponeurosporene	15.59		nd			403			1.93	1.92

### 3.4. Scavenging of reactive oxygen species

**Figure 5:**
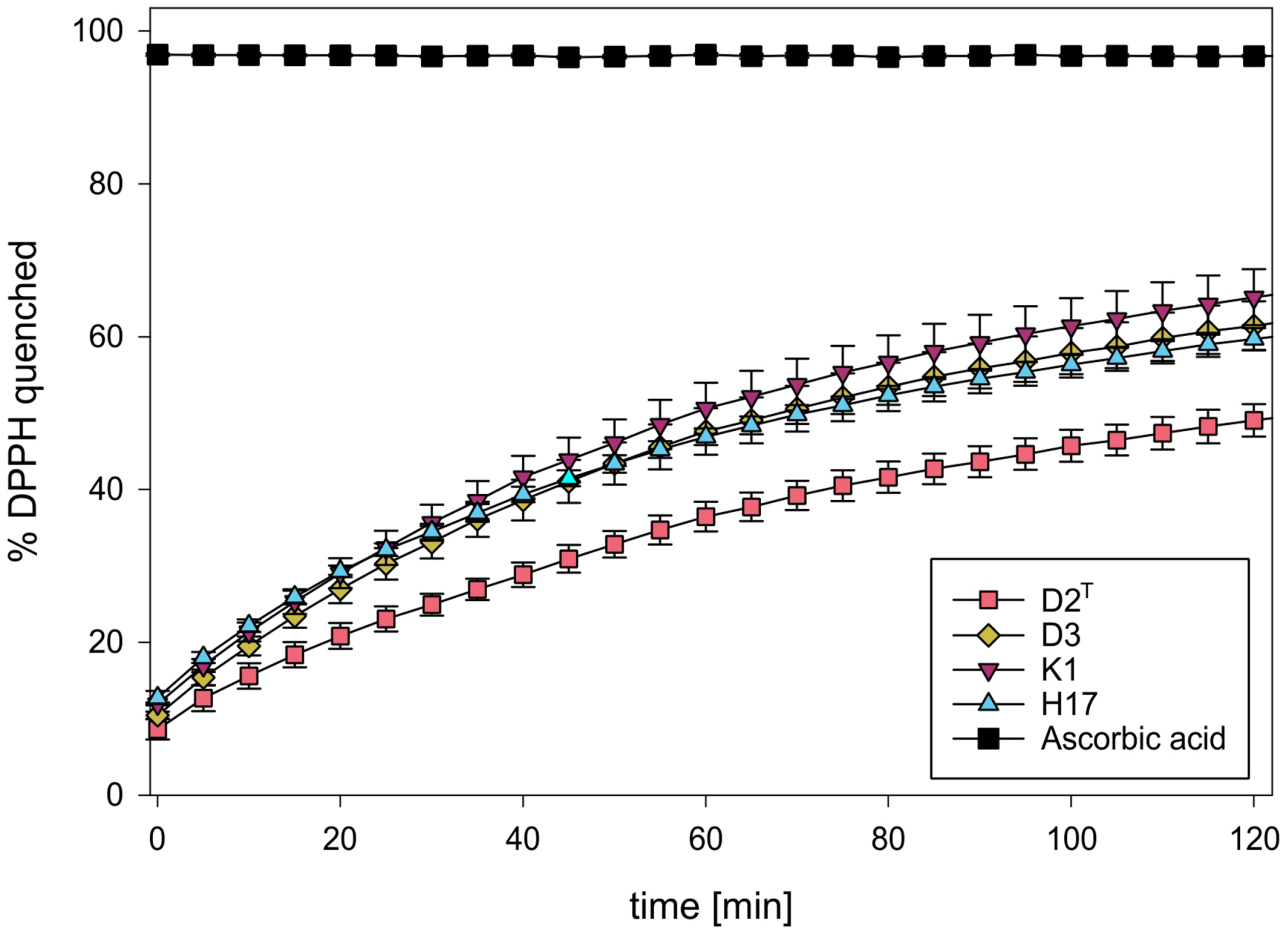
Percentage of scavenged DPPH during incubation with methanol extracts of *S. capitis* subsp. *capitis* strains D2^T^ (red), D3 (yellow), K1 (violet), H17 (blue). Ascorbic acid (5 mg/mL) was used as positive control. Experiment was performed in triplicates. Error bars represent standard error.

The ROS scavenging capacity of methanol cell extracts was detected by the decrease in absorbance of DPPH, at 517 nm (Supplementary Figure S11). Figure 5 shows the calculated average percentage of scavenged DPPH according to the measured absorption values. All four strains showed an increase in the amount of scavenged DPPH over time whereby the unpigmented extract of strain D2^T^ showed the slowest scavenging activity and reached about 50% scavenged DPPH after 120 min. The other three strains showed similar scavenging activity with a slightly increased scavenging activity of strain K1, reaching a maximum scavenging activity of 65% after 120 min.

### 3.5. Survival in temperature stress

**Figure 6:**
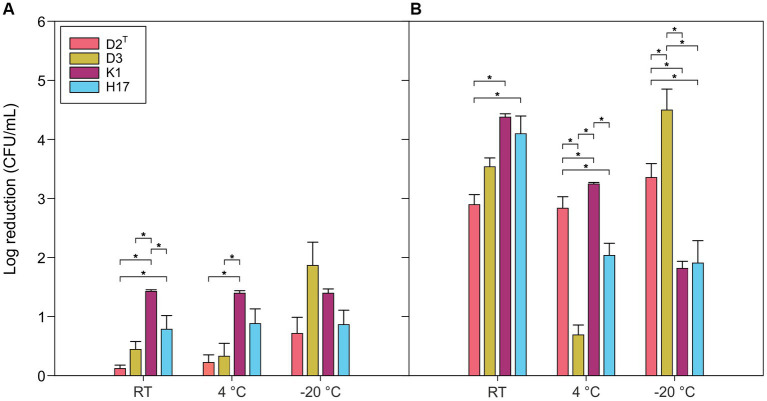
Log reduction of CFU/mL of *S. capitis* subsp. *capitis* strains D2^T^ (red), D3 (yellow), K1 (violet), H17 (blue) after storage in PBS at RT (RT 20°C +/− 2°C), in the fridge (4°C) and in the freezer (−20°C) after 1 day and **(A)** and additional 43 days [=44 days, **(B)**]. Experiment was performed in biological triplicates. Error bars represent the calculated standard error. Asterisks indicate statistical significance (*p* < 0.05).

Figure 6 shows the log reduction of CFU/mL of the four strains after 1 day and 44 days at 4°C, −20°C and RT. After 1 day, D2^T^ and D3 only showed minor reduction in CFU/mL at RT and 4°C whereas K1 and especially H17 showed a larger reduction. At −20°C the CFU/mL decreased in strains D2^T^ and D3 but remained at the same level for the other two strains with respect to the other temperatures. Here, D3 showed the largest reduction in CFU/mL. After 44 days of storage, further log reduction occurred in all four strains. D2^T^ showed similar reduction for all three tested temperatures, whereas the other strains showed a more varied response to the different temperatures. D3 showed high survival in 4°C but a high log reduction for RT and −20°C. The two pigment-producing strains (K1, H17) showed significantly higher survival to −20°C after 44 days than the other two strains. However, at RT both pigment-producing strains showed a slightly higher reduction. At 4°C, strain K1 showed the most reduction whereas strain H17 survived better than strain D2^T^. Incubation of the four strains for 1 hour at 37°C, 50°C and 60°C revealed a high reduction of CFU/mL at 50°C (Supplementary Figure S12). After 1 hour at 60°C, no CFU/mL were detected for any of the strains.

### 3.6. Genetic analysis *crt* operon

**Figure 7:**
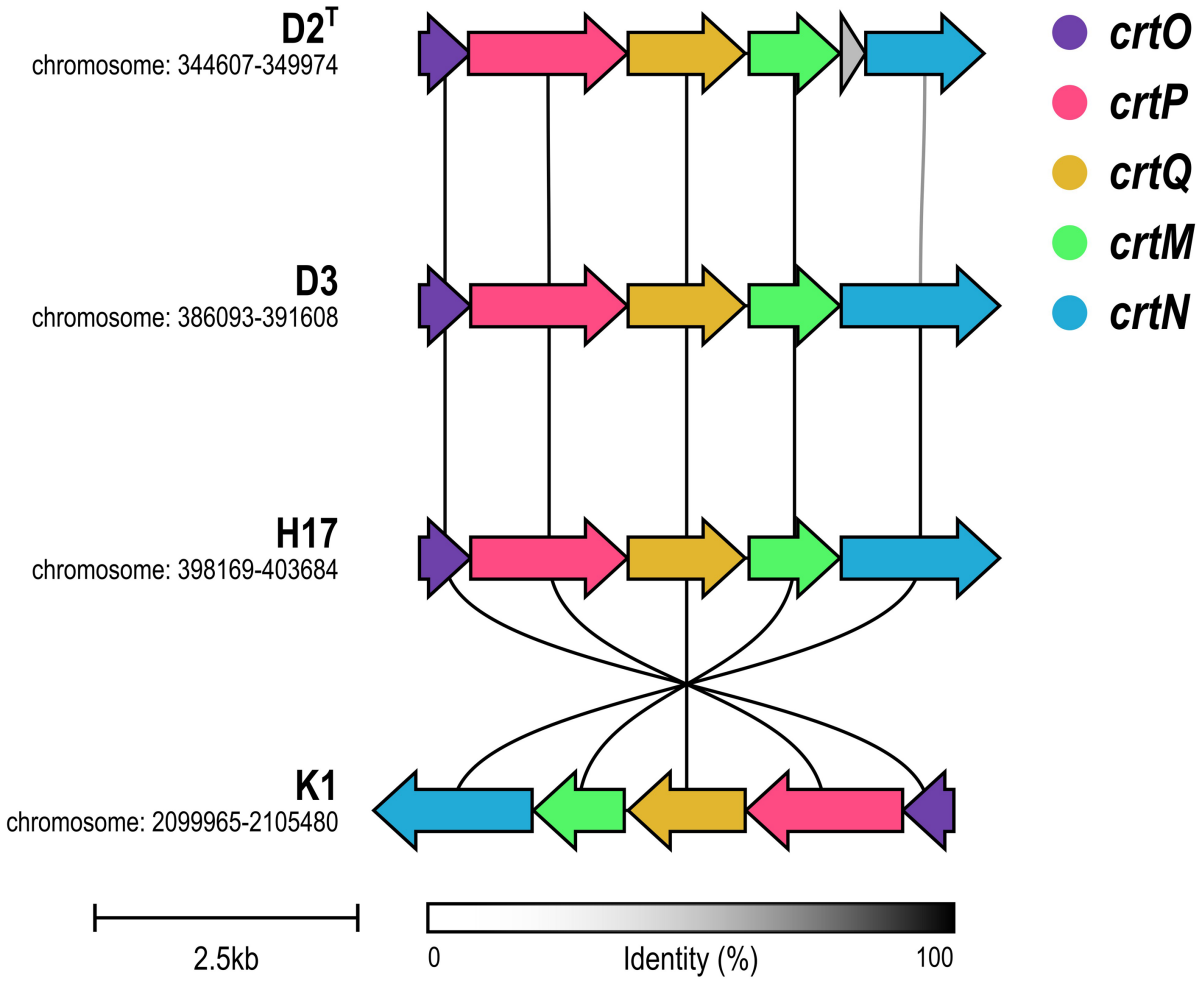
Synteny of *crtOPQMN* operon in *S. capitis* subsp. *capitis* strains. Genes are visualized in colors according to the figure legend. Color of connecting lines indicates sequence identity.

The cluster for the biosynthesis of the carotenoid staphyloxanthin and its precursors was identified by annotation of the biosynthetic genes in the *crtOPQMN* operon for all four strains. Figure 7 visualizes the synteny of the operon. All four strains possess the five genes *crtO, crtP, crtQ, crtM, crtM* and *crtN,* that are involved in the biosynthesis pathway of staphyloxanthin (Supplementary Table S3). D2^T^, D3 and H17 show strong synteny, whereas strain K1 shows an inversion of the operon. Except for *crtN* in D2^T^, all genes show a high sequence identity which was confirmed by alignment of the genes on amino acid level (Supplementary Table S4). In D2^T^, *crtN* was predicted as two, separate open reading frames, as depicted in Figure 7 as grey and shortened blue arrow, caused by a frameshift adding new stop codon (Supplementary Table S5).

## 4. Discussion

The subspecies *S. capitis* subsp. *capitis* was first described as unpigmented (Bannerman and Kloos, 1991) whereas 73% of *S. capitis* subsp. *urealyticus* strains show delayed yellow pigmentation which, to our knowledge, was also not identified yet. All strains used for this study were previously classified as *S. capitis* subsp. *capitis* (Siems et al., 2022), however, two (K1 and H17) showed delayed yellow colony pigmentation despite their classification as subsp. *capitis.* Our results show that pigmentation seemed to be dependent on time of incubation as well as type of medium. Pigmentation appeared strongest on low nutrient R2A medium and less prominent on more nutrient rich media such as CBA or TSA. This might indicate, that pigmentation in the two pigmented strains is a response to stress induced by nutrient depletion. It was shown before that pigmentation in other bacterial species can be influenced by environmental stress factors such as pH, temperature, radiation (gamma and UV), oxygen and salt concentrations (Fong et al., 2001; Asker et al., 2007; Dieser et al., 2010; Sajjad et al., 2020; Seel et al., 2020).

Raman spectra of the colonies of the two pigmented strains showed peaks at 1,159 and 1,523 cm^−1^ which correspond to =C–C= and –C=C-bonds of carotenoid pigments, such as staphyloxanthin (Ayala et al., 2018). In strain H17, individual colonies show significant variances in their pigment-associated Raman peaks which could indicate delayed colony pigmentation. In the HPLC-AD-APCI-MS^n^, strains K1 and H17 show similar profiles to *S. xylosus* (Seel et al., 2020). Contrary to that, for the type strain D2^T^ and strain D3 only one peak was detected with an absorbance at 450 nm, but here, concentration and ionization were too low for carotenoid identification. Tentatively identified carotenoids by HPLC-AD-APCI-MS^n^ in the two pigmented strains were all-*trans*-staphyloxanthin, staphyloxanthin-like compounds, and the staphyloxanthin precursors all-*trans*-4,4′-diaponeurosporenoic acid and 4,4′-diaponeurosporene. In total, four staphyloxanthin-like carotenoids were detected. These appear to have the same carotenoid backbone (433 *m*/*z*) and glucose but vary in their fatty acids because of the different retention times. With increasing lengths of the carotenoid fatty acids chains, the retention time increases as a result of the lower polarity. Staphyloxanthin-like compounds have been detected before in other staphylococci (López et al., 2021; Linden et al., 2023). A limitation of our method of identification is that staphyloxanthin is not commercially available as a standard. Because of this, tuning of the MS had to be performed with β-carotene, which makes the identification more difficult due to poor ionization and fragmentation. Nevertheless, our results strongly indicate the presence of staphyloxanthin, its precursors, and staphyloxanthin-like derivates in *S. capitis* subsp. *capitis* strains K1 and H17. Previous studies identified staphyloxanthin via NMR as β-d-glucopyranosyl 1-O-(4,4′-diaponeurosporen-4-oate)-6-O-(12-methyltetradecanoate) which is the golden pigment abundant in the opportunistic human pathogen *S. aureus* (Pelz et al., 2005). In *S. aureus*, staphyloxanthin represents an important virulence factor which provides protection from ROS through its chemical structure (Liu et al., 2005). Additionally, it stabilizes the cell membrane and therefore modulates membrane fluidity (Mishra et al., 2011). Furthermore, pigment-producing methicillin-resistant *S. aureus* strains seem to be more resistant to drying compared to non-pigmented strains (Beard-Pegler et al., 1988). In a previous study, we observed that the two pigmented strains were more resistant to H_2_O_2_ compared to the two non-pigmented strains but highest tolerance to desiccation was observed in the non-pigmented strain, D3 (Siems et al., 2022). In this study, we saw that both pigmented strains showed ROS scavenging activity in the DPPH assay. This aligns with the known antioxidant properties of carotenoids, like staphyloxanthin (El-Agamey et al., 2004; Clauditz et al., 2006). More specifically, the high electron density of carotenoid compounds, especially of conjugated C=C double bonds, is responsible for the antioxidative and ROS scavenging activity of carotenoids which occurs through physical or chemical reactions (Siziya et al., 2022). This is why, pre-cursors of staphyloxanthin, such as the yellow compounds 4,4′-diaponeurosporene and 4,4′-diaponeurosporenoic acid, that also contain conjugated double bonds, exhibit antioxidative properties (Kim et al., 2016, 2023). In the DPPH assay, the two unpigmented strains also showed ROS scavenging whereby scavenging activity in D2^T^ was lower. This indicates ROS scavenging activity of colourless compounds from the methanol cell extracts. Since we used wildtype strains and no knockout mutant strains, the effect between pigmented and non-pigmentated strains might be less clear due to the occurrence of colorless precursor molecules of staphyloxanthin such as dehydrosqualene (4,4′-diapophytoene). Besides the lack of color, this precursor has fewer conjugated double bonds than staphyloxanthin, 4,4′-diaponeurosporene or 4,4′-diaponeurosporenoic acid (Siziya et al., 2022), but depending on the concentration of the compund in the cell still have an antioxidative effect. Furthermore, we cannot exclude the influence of other methanol-soluble compounds in the methanol extracts from the *S. capitis* cell pellets on ROS scavenging.

A previous study showed that carotenoids, such as staphyloxanthin, regulate membrane fluidity allowing cold adaptation in *S. xylosus,* thus leading to higher survival during multiple freeze-thaw cycles (Seel et al., 2020). In our study, the strains were stored for 1 day and subsequent 44 days at −20°C and therefore exposed to a maximum of two freeze-thaw cycles. We observed that the two pigment-producing strains had consistent reduction in viability after 1 day regardless of temperature, however had a higher survival rate at −20°C after 44 days of storage compared to the non-pigmented strains. Interestingly, the clean room isolate, D3 shows a high tolerance to storage at 4°C which might correlate with its generally increased robustness to environmental stress conditions (Siems et al., 2022).

On a genomic level, the *crtOPQMN* cluster encodes the genes involved in the biosynthesis of staphyloxanthin and its precursors (Pelz et al., 2005). A recent study found that the biosynthetic gene cluster for staphyloxanthin is ubiquitous throughout the *Staphylococcus* genus including *S. capitis* (Salamzade et al., 2023). This aligns with other studies that detected the *crtOPQMN* operon either completely (Watanabe et al., 2018) or in parts (Chong et al., 2022), in *S. capitis*. However, staphyloxanthin itself has never been identified in *S. capitis* prior to this study. We found that all four strains have the complete *crt* operon but type strain D2^T^ shows a deletion of two nucleotides, thus introducing of a stop codon into *crtN,* which codes for a dehydrosqualene desaturase, that catalyzes the formation of the yellow intermediate, 4,4′-diaponeurosporene (CrtN) (Pelz et al., 2005). It was shown before in *S. aureus,* that only strains with intact *crtM* and *crtN* genes are able to produce the deep-yellow staphyloxanthin intermediate 4,4′-diaponeurosporene (Wieland et al., 1994). This could be the reason for the lack of pigmentation observed in the type strain, but cannot explain lack of pigmentation in D3 which shows no significant differences from the pigmented strains within the *crtOPQMN* operon. However, lack of pigmentation in the non-pigmentated strains could also be due to differences in gene regulation. For *S. aureus,* several genes and gene clusters are known to be involved in the biosynthesis of staphyloxanthin, one of them being *rsbUVWsigB* (Palma and Cheung, 2001). In strain K1, the whole *crtOPQMN* operon is inverted which seems to have no effect on pigment production. Merrikh and Merrikh (2018) suggest that gene inversions from the leading strand to the lagging strand lead to a higher gene-specific mutation rate and accelerated evolution through head on replication-transcription conflicts, which could be a beneficial mechanism in bacterial stress response and includes in particular genes encoding virulence factors, antibiotic resistance proteins or transcription regulators.

We conclude that the identification of staphyloxanthin in *S. capitis* subsp. *capitis* is a significant discovery with clinical relevance as staphyloxanthin is a well-known virulence factor in *S. aureus*, which protects cells from ROS stress and modulates membrane fluidity. ROS scavenging activity and increased adaptation to cold temperature were partially confirmed for *S. capitis* subsp. *capitis* in this study. All four strains showed ROS scavenging activity regardless of staphyloxanthin production indicating an influence of other antioxidative compounds such as staphyloxanthin precursor molecules. Furthermore, an increased survival to cold temperatures was detected after long-term storage. The identification of staphyloxanthin in *S. capitis* subsp. *capitis* could be used in the same way as in *S. aureus* as a possible target for anti-virulence drug design (Xue et al., 2019) especially when considering the emergence of antibiotic resistant strains in clinical settings.

## Data availability statement

The datasets presented in this study can be found in online repositories. The names of the repository/repositories and accession number(s) can be found in the article/Supplementary material.

## Author contributions

KS: Conceptualization, Investigation, Writing – original draft, Writing – review & editing. KRu: Conceptualization, Investigation, Writing – review & editing. KRe: Conceptualization, Investigation, Writing – review & editing. LE: Conceptualization, Investigation, Writing – review & editing. AA: Investigation, Writing – review & editing. AR: Conceptualization, Investigation, Writing – review & editing. OSc: Conceptualization, Investigation, Writing – review & editing. MŠ: Investigation, Writing – review & editing. OSa: Supervision, Writing – review & editing. FR: Supervision, Writing – review & editing. RM: Conceptualization, Investigation, Supervision, Writing – review & editing.

## Funding

The author(s) declare financial support was received for the research, authorship, and/or publication of this article. KS and RM were supported by the DLR grant FuE-Projekt “ISS LIFE” (Programm RF-FuW, Teilprogramm 475). KRe and FR were supported by the Grant Agency of Masaryk University (MUNI/A/1361/2022). KRe, FR, OSc, and MŠ were supported by the Czech Health Research Council (NU21-05-00341) grant and ISI CAS.

## Conflict of interest

The authors declare that the research was conducted in the absence of any commercial or financial relationships that could be construed as a potential conflict of interest.

## Publisher’s note

All claims expressed in this article are solely those of the authors and do not necessarily represent those of their affiliated organizations, or those of the publisher, the editors and the reviewers. Any product that may be evaluated in this article, or claim that may be made by its manufacturer, is not guaranteed or endorsed by the publisher.

## References

[ref1] Asker D. Beppu T. Ueda K. (2007). Unique diversity of carotenoid-producing bacteria isolated from Misasa, a radioactive site in Japan. Appl. Microbiol. Biotechnol. 77, 383–392. doi: 10.1007/s00253-007-1157-8, PMID: 17828533

[ref2] Ayala O. D. Wakeman C. A. Pence I. J. Gaddy J. A. Slaughter J. C. Skaar E. P.. (2018). Drug-resistant *Staphylococcus aureus* strains reveal distinct biochemical features with Raman microspectroscopy. ACS Infect. Dis. 4, 1197–1210. doi: 10.1021/acsinfecdis.8b00029, PMID: 29845863 PMC6476553

[ref3] Bannerman T. L. Kloos W. E. (1991). *Staphylococcus capitis* subsp. *ureolyticus* subsp. nov. from human skin. Int. J. Syst. Bacteriol. 41, 144–147. doi: 10.1099/00207713-41-1-144, PMID: 1995030

[ref4] Beard-Pegler M. A. Stubbs E. Vickery A. M. (1988). Observations on the resistance to drying of staphylococcal strains. J. Med. Microbiol. 26, 251–255. doi: 10.1099/00222615-26-4-251, PMID: 3398031

[ref5] Bernatova S. Samek O. Pilat Z. Sery M. Jezek J. Jakl P.. (2013). Following the mechanisms of bacteriostatic versus bactericidal action using Raman spectroscopy. Molecules 18, 13188–13199. doi: 10.3390/molecules181113188, PMID: 24284484 PMC6270526

[ref6] Breithaupt D. E. Schwack W. Wolf G. Hammes W. P. (2001). Characterization of the triterpenoid 4,4′-diaponeurosporene and its isomers in food-associated bacteria. Eur. Food Res. Technol. 213, 231–233. doi: 10.1007/s002170100358

[ref7] Bull A. T. (1970). Inhibition of polysaccharases by melanin: enzyme inhibition in relation to mycolysis. Arch. Biochem. Biophys. 137, 345–356. doi: 10.1016/0003-9861(70)90448-0, PMID: 4191418

[ref8] Cameron D. R. Jiang J. H. Hassan K. A. Elbourne L. D. Tuck K. L. Paulsen I. T.. (2015). Insights on virulence from the complete genome of *Staphylococcus capitis*. Front. Microbiol. 6:980. doi: 10.3389/fmicb.2015.00980, PMID: 26441910 PMC4585213

[ref9] Chong C. E. Bengtsson R. J. Horsburgh M. J. (2022). Comparative genomics of *Staphylococcus capitis* reveals species determinants. Front. Microbiol. 13:1005949. doi: 10.3389/fmicb.2022.1005949, PMID: 36246238 PMC9563023

[ref10] Clauditz A. Resch A. Wieland K. P. Peschel A. Götz F. (2006). Staphyloxanthin plays a role in the fitness of *Staphylococcus aureus* and its ability to cope with oxidative stress. Infect. Immun. 74, 4950–4953. doi: 10.1128/IAI.00204-06, PMID: 16861688 PMC1539600

[ref11] Cui B. Smooker P. M. Rouch D. A. Daley A. J. Deighton M. A. (2013). Differences between two clinical *Staphylococcus capitis* subspecies as revealed by biofilm, antibiotic resistance, and pulsed-field gel electrophoresis profiling. J. Clin. Microbiol. 51, 9–14. doi: 10.1128/JCM.05124-11, PMID: 23052315 PMC3536240

[ref12] De Maesschalck R. Jouan-Rimbaud D. Massart D. L. (2000). The Mahalanobis distance. Chemom. Intell. Lab. Syst. 50, 1–18. doi: 10.1016/S0169-7439(99)00047-7, PMID: 37671618

[ref13] Decalonne M. Dos Santos S. Gimenes R. Goube F. Abadie G. Aberrane S.. (2020). *Staphylococcus capitis* isolated from bloodstream infections: a nationwide 3-month survey in 38 neonatal intensive care units. Eur. J. Clin. Microbiol. Infect. Dis. 39, 2185–2194. doi: 10.1007/s10096-020-03925-5, PMID: 32519215 PMC7561542

[ref14] Dieckmann M. A. B. S. Nkouamedjo-Fankep R. C. Hanel P. H. G. Jelonek L. Blom J. Goesmann A. (2021). EDGAR3.0: comparative genomics and phylogenomics on a scalable infrastructure. Nucleic Acids Res. 49, W185–W192. doi: 10.1093/nar/gkab341, PMID: 33988716 PMC8262741

[ref15] Dieser M. Greenwood M. Foreman C. M. (2010). Carotenoid pigmentation in Antarctic heterotrophic bacteria as a strategy to withstand environmental stresses. Arct. Antarct. Alp. Res. 42, 396–405. doi: 10.1657/1938-4246-42.4.396

[ref16] Edgar R. C. (2004). MUSCLE: multiple sequence alignment with high accuracy and high throughput. Nucleic Acids Res. 32, 1792–1797. doi: 10.1093/nar/gkh340, PMID: 15034147 PMC390337

[ref17] El-Agamey A. Lowe G. M. McGarvey D. J. Mortensen A. Phillip D. M. Truscott T. G.. (2004). Carotenoid radical chemistry and antioxidant/pro-oxidant properties. Arch. Biochem. Biophys. 430, 37–48. doi: 10.1016/j.abb.2004.03.007, PMID: 15325910

[ref18] Etzbach L. Pfeiffer A. Weber F. Schieber A. (2018). Characterization of carotenoid profiles in goldenberry (*Physalis peruviana* L.) fruits at various ripening stages and in different plant tissues by HPLC-DAD-APCI-MSn. Food Chem. 245, 508–517. doi: 10.1016/j.foodchem.2017.10.120 29287402

[ref19] Fong N. Burgess M. Barrow K. Glenn D. (2001). Carotenoid accumulation in the psychrotrophic bacterium *Arthrobacter agilis* in response to thermal and salt stress. Appl. Microbiol. Biotechnol. 56, 750–756. doi: 10.1007/s002530100739 11601625

[ref20] Gilchrist C. L. M. Chooi Y.-H. (2021). clinker & clustermap.js: automatic generation of gene cluster comparison figures. Bioinformatics 37, 2473–2475. doi: 10.1093/bioinformatics/btab007, PMID: 33459763

[ref21] Kaiser P. Surmann P. Vallentin G. Fuhrmann H. (2007). A small-scale method for quantitation of carotenoids in bacteria and yeasts. J. Microbiol. Methods 70, 142–149. doi: 10.1016/j.mimet.2007.04.004, PMID: 17509707

[ref22] Kim M. Jung D. H. Hwang C. Y. Siziya I. N. Park Y. S. Seo M. J. (2023). 4,4′-diaponeurosporene production as C30 carotenoid with antioxidant activity in recombinant *Escherichia coli*. Appl. Biochem. Biotechnol. 195, 135–151. doi: 10.1007/s12010-022-04147-5, PMID: 36066805

[ref23] Kim S. H. Kim M. S. Lee B. Y. Lee P. C. (2016). Generation of structurally novel short carotenoids and study of their biological activity. Sci. Rep. 6, 1–11. doi: 10.1038/srep21987, PMID: 26902326 PMC4763220

[ref24] Linden M. Flegler A. Feuereisen M. M. Weber F. Lipski A. Schieber A. (2023). Effects of flavonoids on membrane adaptation of food-associated bacteria. Biochim. Biophys. Acta 1865:184137. doi: 10.1016/j.bbamem.2023.184137, PMID: 36746312

[ref25] Liu G. Y. Essex A. Buchanan J. T. Datta V. Hoffman H. M. Bastian J. F.. (2005). *Staphylococcus aureus* golden pigment impairs neutrophil killing and promotes virulence through its antioxidant activity. J. Exp. Med. 202, 209–215. doi: 10.1084/jem.20050846, PMID: 16009720 PMC2213009

[ref26] López G. D. Suesca E. Álvarez-Rivera G. Rosato A. E. Ibáñez E. Cifuentes A.. (2021). Carotenogenesis of *Staphylococcus aureus*: new insights and impact on membrane biophysical properties. Biochim. Biophys. Acta 1866:158941. doi: 10.1016/j.bbalip.2021.158941, PMID: 33862238

[ref27] Madeira F. Pearce M. Tivey A. R. N. Basutkar P. Lee J. Edbali O.. (2022). Search and sequence analysis tools services from EMBL-EBI in 2022. Nucleic Acids Res. 50, W276–W279. doi: 10.1093/nar/gkac240, PMID: 35412617 PMC9252731

[ref28] Marshall J. H. Wilmoth G. J. (1981). Pigments of *Staphylococcus aureus*, a series of triterpenoid carotenoids. J. Bacteriol. 147, 900–913. doi: 10.1128/jb.147.3.900-913.1981, PMID: 7275936 PMC216126

[ref29] Merrikh C. N. Merrikh H. (2018). Gene inversion potentiates bacterial evolvability and virulence. Nat. Commun. 9:4662. doi: 10.1038/s41467-018-07110-3 30405125 PMC6220195

[ref30] Mishra N. N. Liu G. Y. Yeaman M. R. Nast C. C. Proctor R. A. McKinnell J.. (2011). Carotenoid-related alteration of cell membrane fluidity impacts *Staphylococcus aureus* susceptibility to host defense peptides. Antimicrob. Agents Chemother. 55, 526–531. doi: 10.1128/AAC.00680-10, PMID: 21115796 PMC3028772

[ref31] Mlynarikova K. Samek O. Bernatova S. Ruzicka F. Jezek J. Haronikova A.. (2015). Influence of culture media on microbial fingerprints using Raman spectroscopy. Sensors 15, 29635–29647. doi: 10.3390/s151129635, PMID: 26610516 PMC4701351

[ref32] Moore J. Yu L. (2007). “Methods for antioxidant capacity estimation of wheat and wheat-based food products” in Wheat antioxidants. ed. Yu L. (Hoboken, New Jersey, United States: John Wiley & Sons, Inc.), 118–172.

[ref33] Mora M. Wink L. Kögler I. Mahnert A. Rettberg P. Schwendner P.. (2019). Space station conditions are selective but do not alter microbial characteristics relevant to human health. Nat. Commun. 10:3990. doi: 10.1038/s41467-019-11682-z 31488812 PMC6728350

[ref34] Natsis N. E. Cohen P. R. (2018). Coagulase-negative *Staphylococcus* skin and soft tissue infections. Am. J. Clin. Dermatol. 19, 671–677. doi: 10.1007/s40257-018-0362-9, PMID: 29882122

[ref35] Palma M. Cheung A. L. (2001). ς^B^ activity in *Staphylococcus aureus* is controlled by RsbU and an additional factor(s) during bacterial growth. Infect. Immun. 69, 7858–7865. doi: 10.1128/IAI.69.12.7858-7865.2001, PMID: 11705968 PMC98882

[ref36] Pannu M. K. Hudman D. A. Sargentini N. J. Singh V. K. (2019). Role of SigB and staphyloxanthin in radiation survival of *Staphylococcus aureus*. Curr. Microbiol. 76, 70–77. doi: 10.1007/s00284-018-1586-x, PMID: 30353215

[ref37] Pelz A. Wieland K.-P. Putzbach K. Hentschel P. Albert K. Götz F. (2005). Structure and biosynthesis of staphyloxanthin from *Staphylococcus aureus*. J. Biol. Chem. 280, 32493–32498. doi: 10.1074/jbc.M505070200, PMID: 16020541

[ref38] Rebrošová K. Šiler M. Samek O. Růžička F. Bernatová S. Ježek J.. (2017). Differentiation between *Staphylococcus aureus* and *Staphylococcus epidermidis* strains using Raman spectroscopy. Future Microbiol. 12, 881–890. doi: 10.2217/fmb-2016-0224, PMID: 28686040

[ref39] Renishaw (2003). Technical note: Renishaw’s EasyConfocal Raman method SPD/TN/076. Wotton-under-Edge. Rehnishaw PLC

[ref40] Sajjad W. Din G. Rafiq M. Iqbal A. Khan S. Zada S.. (2020). Pigment production by cold-adapted bacteria and fungi: colorful tale of cryosphere with wide range applications. Extremophiles 24, 447–473. doi: 10.1007/s00792-020-01180-2, PMID: 32488508 PMC7266124

[ref41] Salamzade R. Cheong J. Z. A. Sandstrom S. Swaney M. H. Stubbendieck R. M. Starr N. L.. (2023). Evolutionary investigations of the biosynthetic diversity in the skin microbiome using *lsa* BGC. Microb. Genom. 9:mgen000988. doi: 10.1099/mgen.0.000988, PMID: 37115189 PMC10210951

[ref42] Schwengers O. Jelonek L. Dieckmann M. A. Beyvers S. Blom J. Goesmann A. (2021). Bakta: rapid and standardized annotation of bacterial genomes via alignment-free sequence identification. Microb. Genom. 7:000685. doi: 10.1099/mgen.0.000685, PMID: 34739369 PMC8743544

[ref43] Seel W. Baust D. Sons D. Albers M. Etzbach L. Fuss J.. (2020). Carotenoids are used as regulators for membrane fluidity by *Staphylococcus xylosus*. Sci. Rep. 10:330. doi: 10.1038/s41598-019-57006-5, PMID: 31941915 PMC6962212

[ref44] Sendra J. M. Sentandreu E. Navarro J. L. (2006). Reduction kinetics of the free stable radical 2,2-diphenyl-1-picrylhydrazyl (DPPH^•^) for determination of the antiradical activity of citrus juices. Eur. Food Res. Technol. 223:615. doi: 10.1007/s00217-005-0243-3

[ref45] Siems K. Runzheimer K. Rehm A. Schwengers O. Heidler von Heilborn D. Kaser L.. (2022). Phenotypic and genomic assessment of the potential threat of human spaceflight-relevant *Staphylococcus capitis* isolates under stress conditions. Front. Microbiol. 13:1007143. doi: 10.3389/fmicb.2022.1007143 36406458 PMC9669719

[ref46] Siziya I. N. Hwang C. Y. Seo M.-J. (2022). Antioxidant potential and capacity of microorganism-sourced C30 carotenoids—a review. Antioxidants 11:1963. doi: 10.3390/antiox11101963, PMID: 36290686 PMC9598406

[ref47] Sobisch L.-Y. Rogowski K. M. Fuchs J. Schmieder W. Vaishampayan A. Oles P.. (2019). Biofilm forming antibiotic resistant gram-positive pathogens isolated from surfaces on the international Space Station. Front. Microbiol. 10:543. doi: 10.3389/fmicb.2019.00543, PMID: 30941112 PMC6433718

[ref48] Verma S. P. Philippot J. R. Bonnet B. Sainte-Marie J. Moschetto Y. Wallach D. F. (1984). Resonance Raman spectra of beta-carotene in native and modified low-density lipoprotein. Biochem. Biophys. Res. Commun. 122, 867–875. doi: 10.1016/S0006-291X(84)80114-X, PMID: 6087818

[ref49] ViroxTechnologies (2018). AHP goes beyond. Available at: https://virox.com/making-news/virox-corp-news/ahp-goes-beyond/ (Accessed August 31, 2023).

[ref50] Watanabe S. Aiba Y. Tan X.-E. Li F.-Y. Boonsiri T. Thitiananpakorn K.. (2018). Complete genome sequencing of three human clinical isolates of *Staphylococcus caprae* reveals virulence factors similar to those of *S. epidermidis* and *S. capitis*. BMC Genomics 19:810. doi: 10.1186/s12864-018-5185-9 30409159 PMC6225691

[ref51] Wieland B. Feil C. Gloria-Maercker E. Thumm G. Lechner M. Bravo J. M.. (1994). Genetic and biochemical analyses of the biosynthesis of the yellow carotenoid 4,4′-diaponeurosporene of *Staphylococcus aureus*. J. Bacteriol. 176, 7719–7726. doi: 10.1128/jb.176.24.7719-7726.1994, PMID: 8002598 PMC197231

[ref52] Wirth T. Bergot M. Rasigade J.-P. Pichon B. Barbier M. Martins-Simoes P.. (2020). Niche specialization and spread of *Staphylococcus capitis* involved in neonatal sepsis. Nat. Microbiol. 5, 735–745. doi: 10.1038/s41564-020-0676-2, PMID: 32341568

[ref53] Xue L. Chen Y. Y. Yan Z. Lu W. Wan D. Zhu H. (2019). Staphyloxanthin: a potential target for antivirulence therapy. Infect. Drug Resist. 12:2151. doi: 10.2147/IDR.S193649 31410034 PMC6647007

[ref54] Yao Z. Y. Qi J. H. (2016). Comparison of antioxidant activities of melanin fractions from chestnut Shell. Molecules 21:487. doi: 10.3390/molecules21040487, PMID: 27110763 PMC6273334

