# Supplementary material for: Identification of staphyloxanthin and derivates in yellow-pigmented Staphylococcus capitis subsp. capitis
Source: Front Microbiol. 2023 Sep 29;14:1272734. doi: 10.3389/fmicb.2023.1272734 (PMC10570620; doi:10.3389/fmicb.2023.1272734)
Supplement: Supplementary file 1 [file Data_Sheet_1.DOCX]

Supplementary Material

# Colony morphology on different media


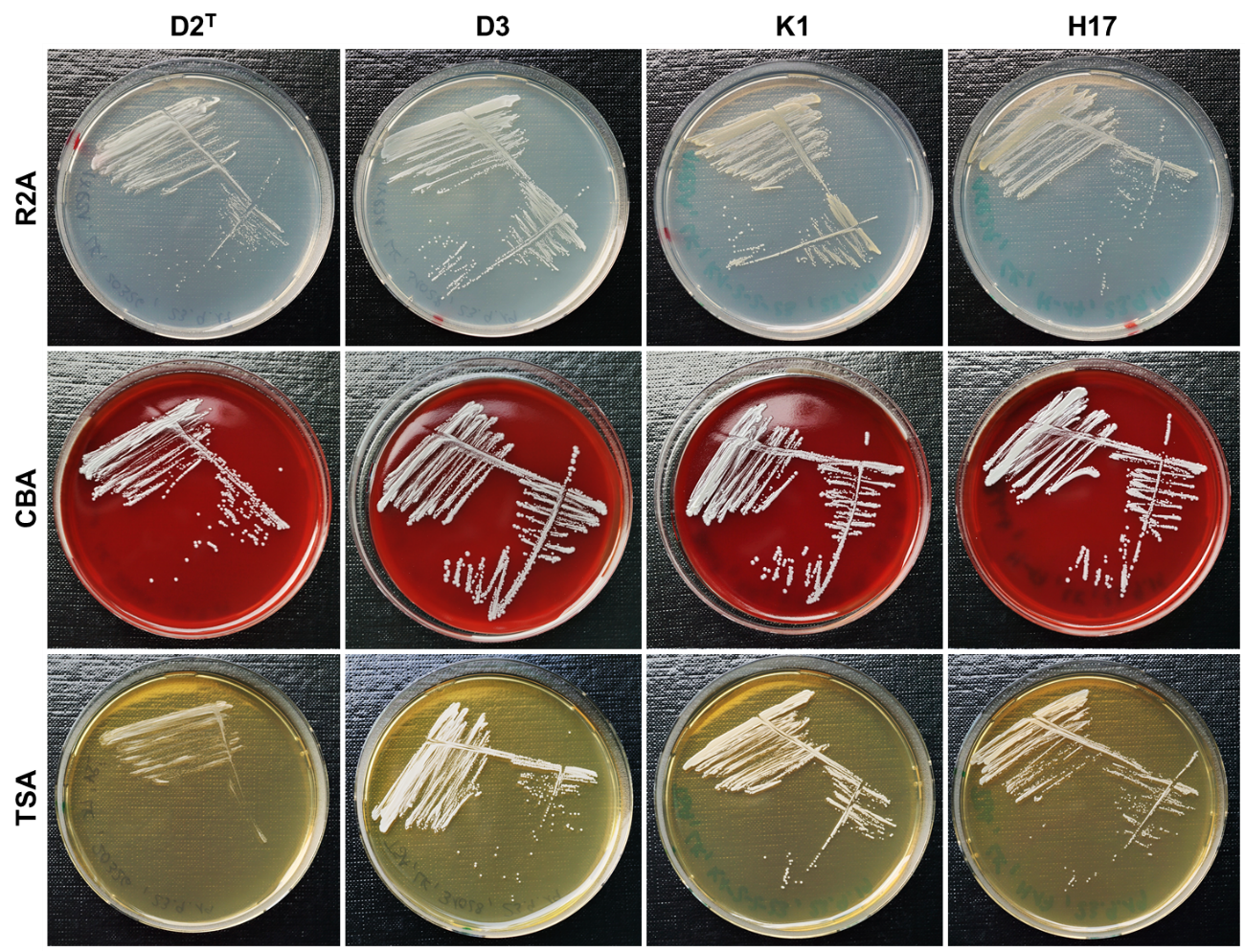


**Supplementary Figure 1** Colony morphology of *S. capitis* subsp. *capitis* strains D2^T^, D3, K1 and H17 cultivated on R2A, CBA and TSA plates (⌀ 9 cm) for 24h at 37 °C. For size reference, diameter of petri dishes is 9 cm.


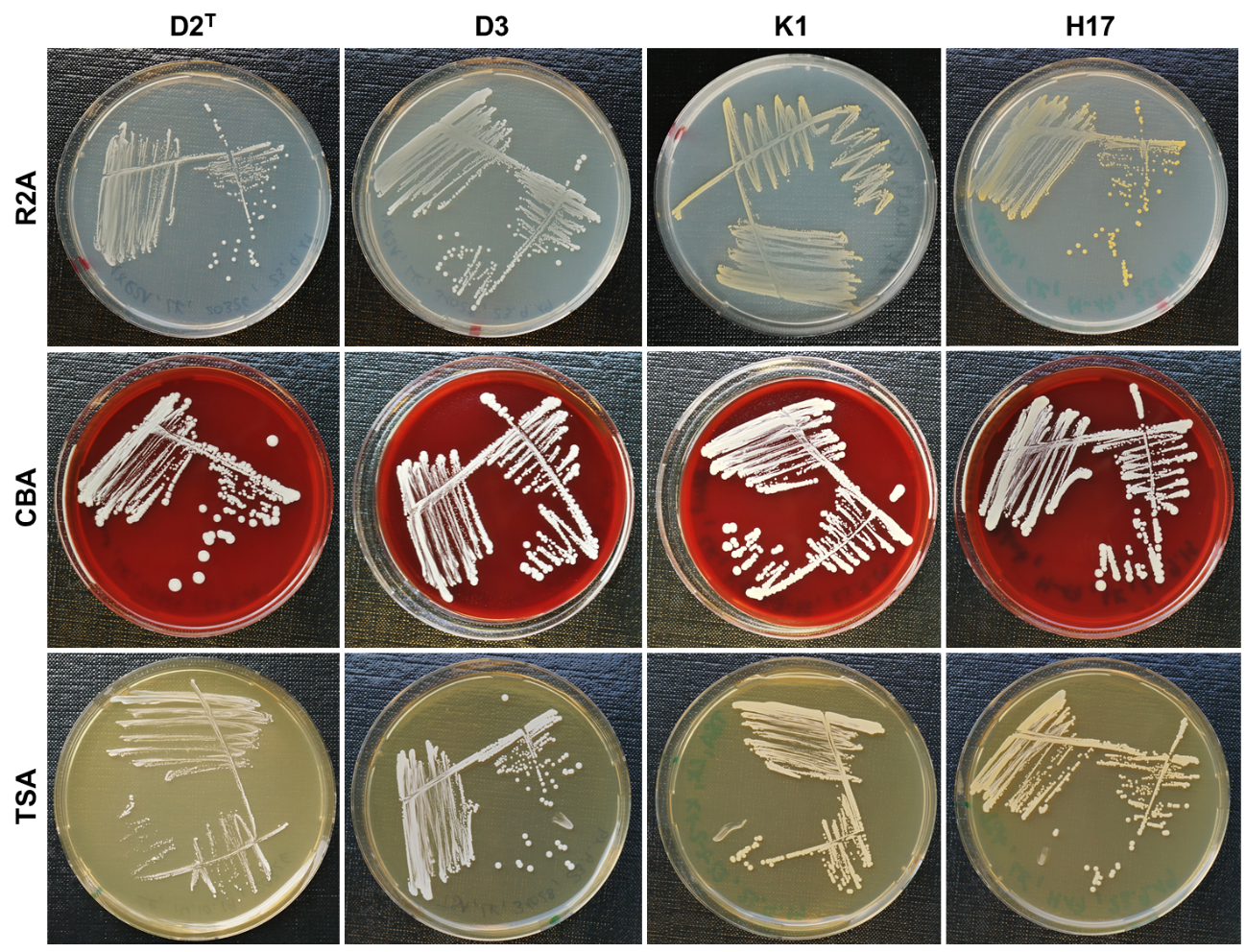


**Supplementary Figure 2** Colony morphology of *S. capitis* subsp. *capitis* strains D2^T^, D3, K1 and H17 cultivated on R2A, CBA and TSA plates (⌀ 9 cm) for a total incubation time of 72h consisting of incubation for 48h at 37 °C and 24h incubation at room temperature.

# Mass Spectrometry

Supplementary Table 1 Parameters and settings for mass spectrometry

| **Parameter** | **Setting** |
| --- | --- |
| Collision gas | Helium |
| Capillary temperature | 275 °C |
| APCI Vaporizer temperature | 400 °C |
| Sheath Gas Flow | 30.00 arb. unit |
| Aux Gas Flow | 5.00 arb. unit |
| Sweep Gas Flow | 5.00 arb. unit |
| Source Voltage | 6.00 kV |
| Source Current | 5.00 µA |
| Capillary Voltage | 15.00 V |
| Tube Lens | 65.00 V |

# Raman spectroscopy

Supplementary Table 2 Suggested Raman peaks assignments according to literature (1-9)

| **Raman Feature (cm^−1^)** | **Suggested Assignment** |
| --- | --- |
| 720 | adenine |
| 780-786 | cytosine, uracil, thymine ring breathing |
| 788 | O-P-O stretch of DNA |
| 813 | O-P-O stretch of RNA |
| 866-898 | C-O-O skeletal vibration of lipids |
| 880 | C-C-N symmetric stretch of lipids |
| 929 | DNA backbone stretching vibration, C-C stretching vibration of proteins |
| 1004 | phenylalanine ring breathing, in-plane rocking vibrations of the C-CH_3_ in carotenoids |
| 1033 | C-H in-plane stretch of Phe |
| 1080 | C-C stretch of lipids |
| 1093 | C-N stretch of proteins |
| 1095 | vibration of phosphodioxy (PO_2_) group |
| 1156, 1523 | carotenoids (staphyloxanthin) |
| 1128 | C-N stretch of proteins |
| 1209 | proteins |
| 1267–1270 | lipids, amide III |
| 1340–1350 | proteins, carbohydrates |
| 1440–1460 | deformation vibration CH_2_ scissoring, lipids |
| 1600–1611 | υ(CC) aromatic ring chain vibrations |
|  | C–C ring stretching—phenylalanine, tyrosine, and tryptophan |
| 1620–1690 | amide I, lipids |


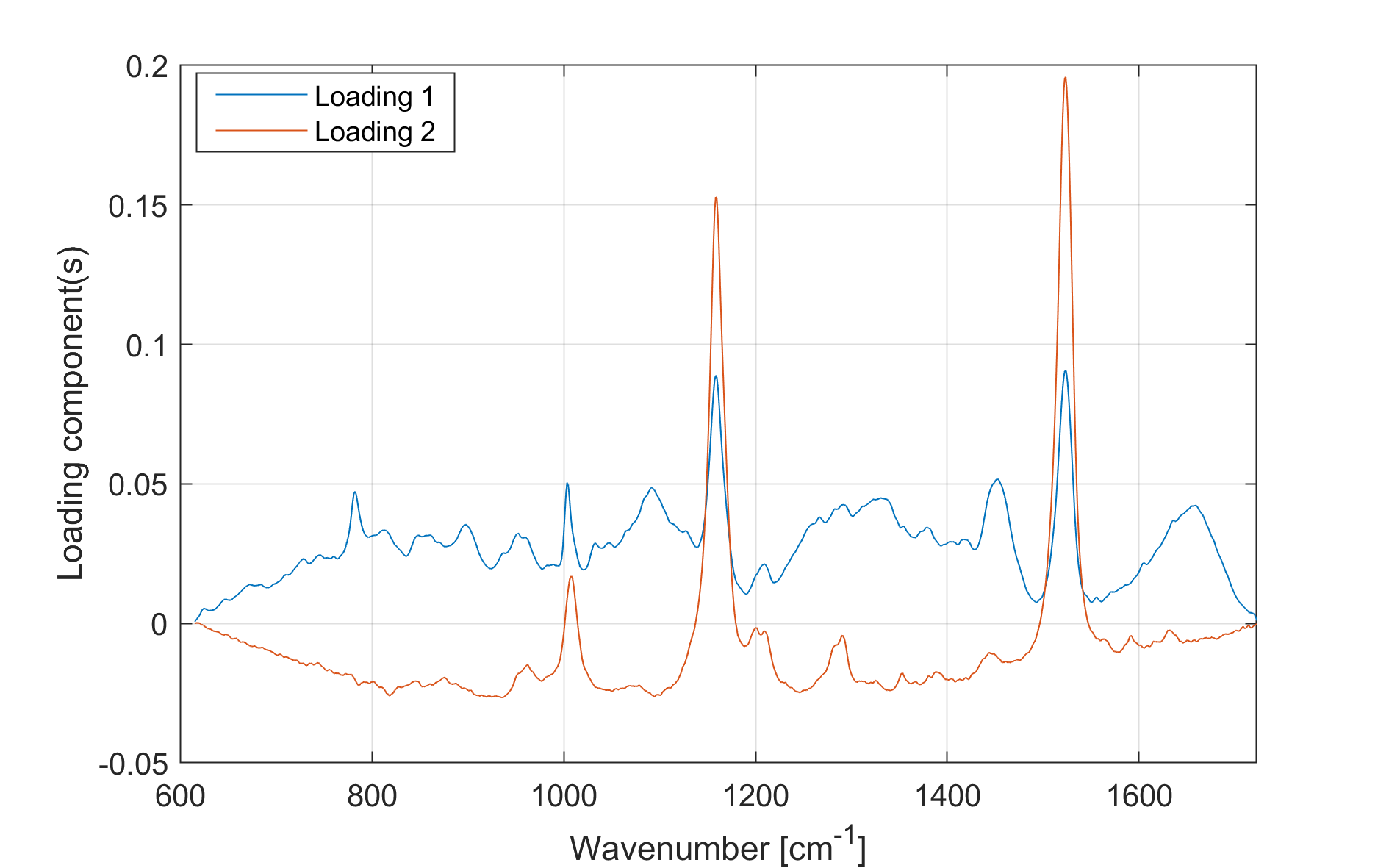


**Supplementary Figure 3** Loadings of the PCA analysis shown in Figure 3 (colonies of four *S. capitis* subsp. *capitis* strains on R2A after incubation at 37 °C for 48h).


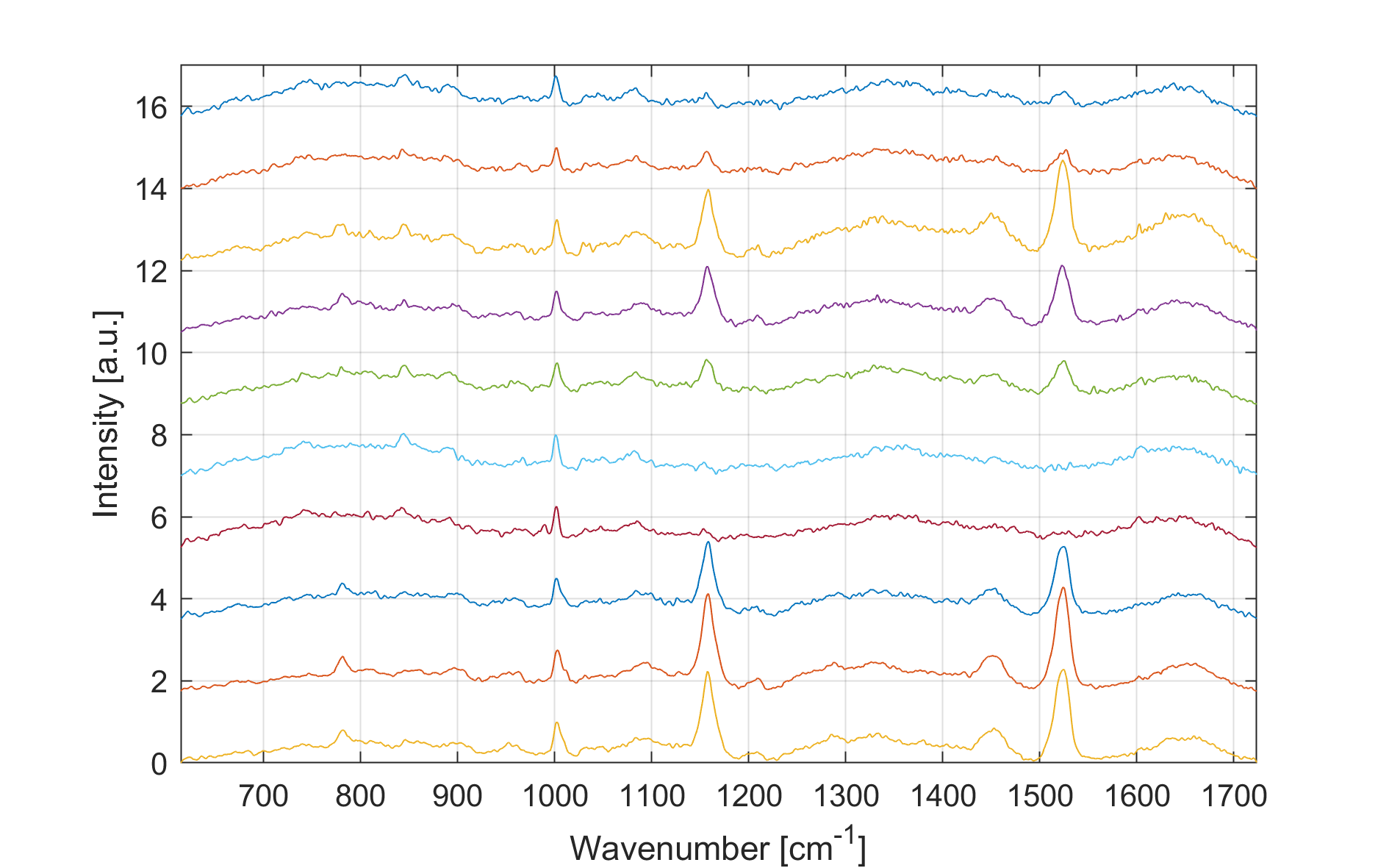


**Supplementary Figure 4** Variability in normalized Raman spectra of *S. capitis* subsp. *capitis* strain H17 on R2A agar after incubation for 24h at 37 °C.


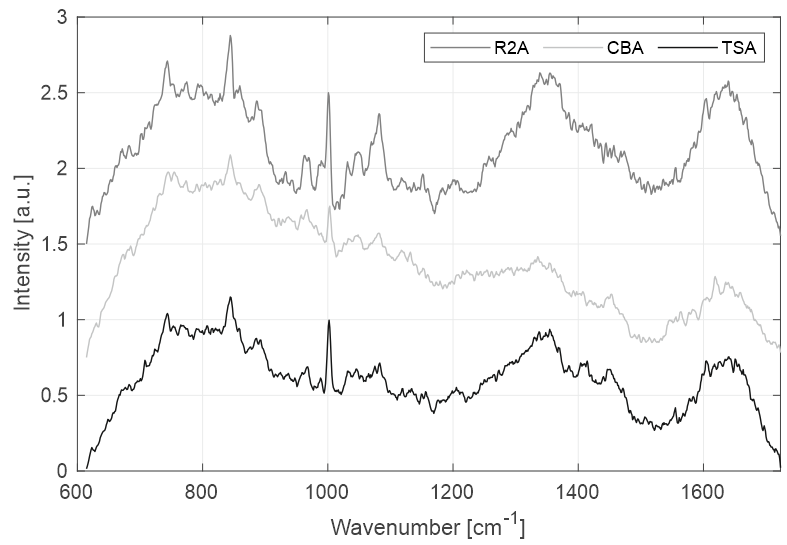


**Supplementary Figure 5** Average Raman spectra of media without bacterial colonies. From top to bottom spectra for TSA (black), R2A (grey), MH (red) and CBA (light grey) are shown.

# Identification of carotenoids


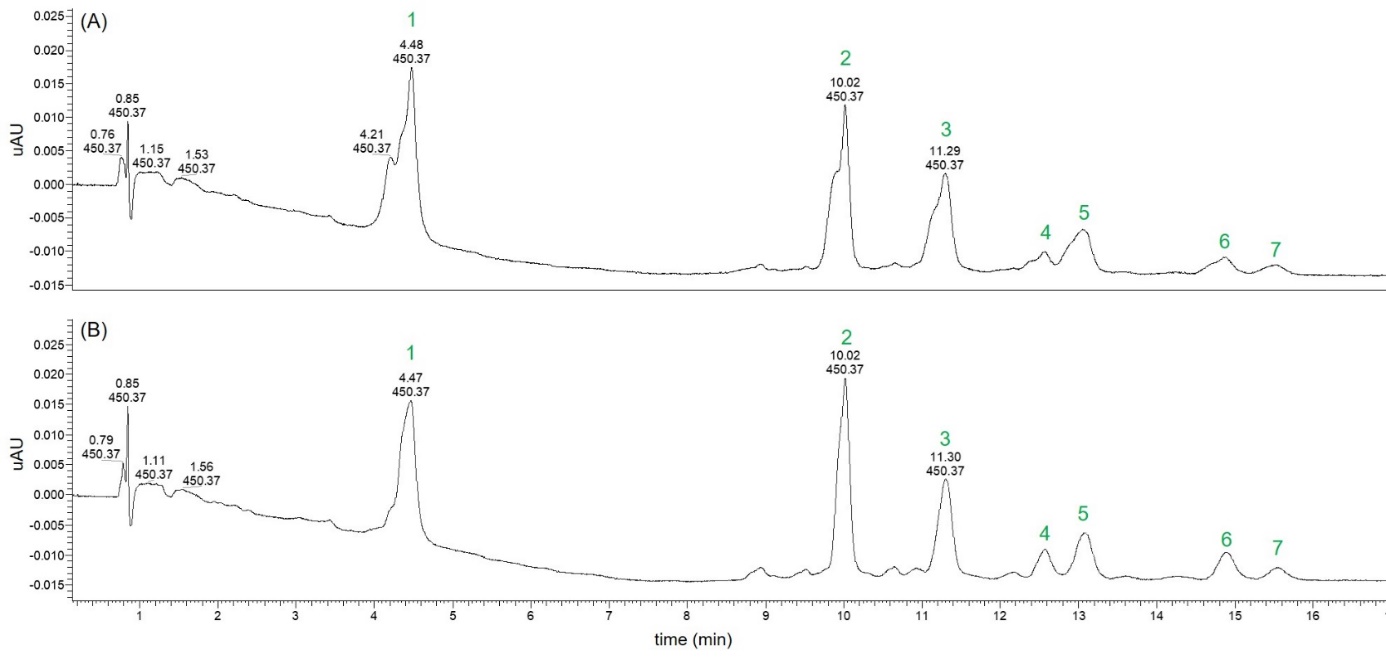


**Supplementary Figure 6** HPLC-DAD-chromatograms (450 nm) of the methanol extracts of *S. capitis* subsp. *capitis* strains K1 (A) and H17 (B). The seven labeled peaks were detected and further identified by mass spectrometry to tentatively identify according carotenoids.


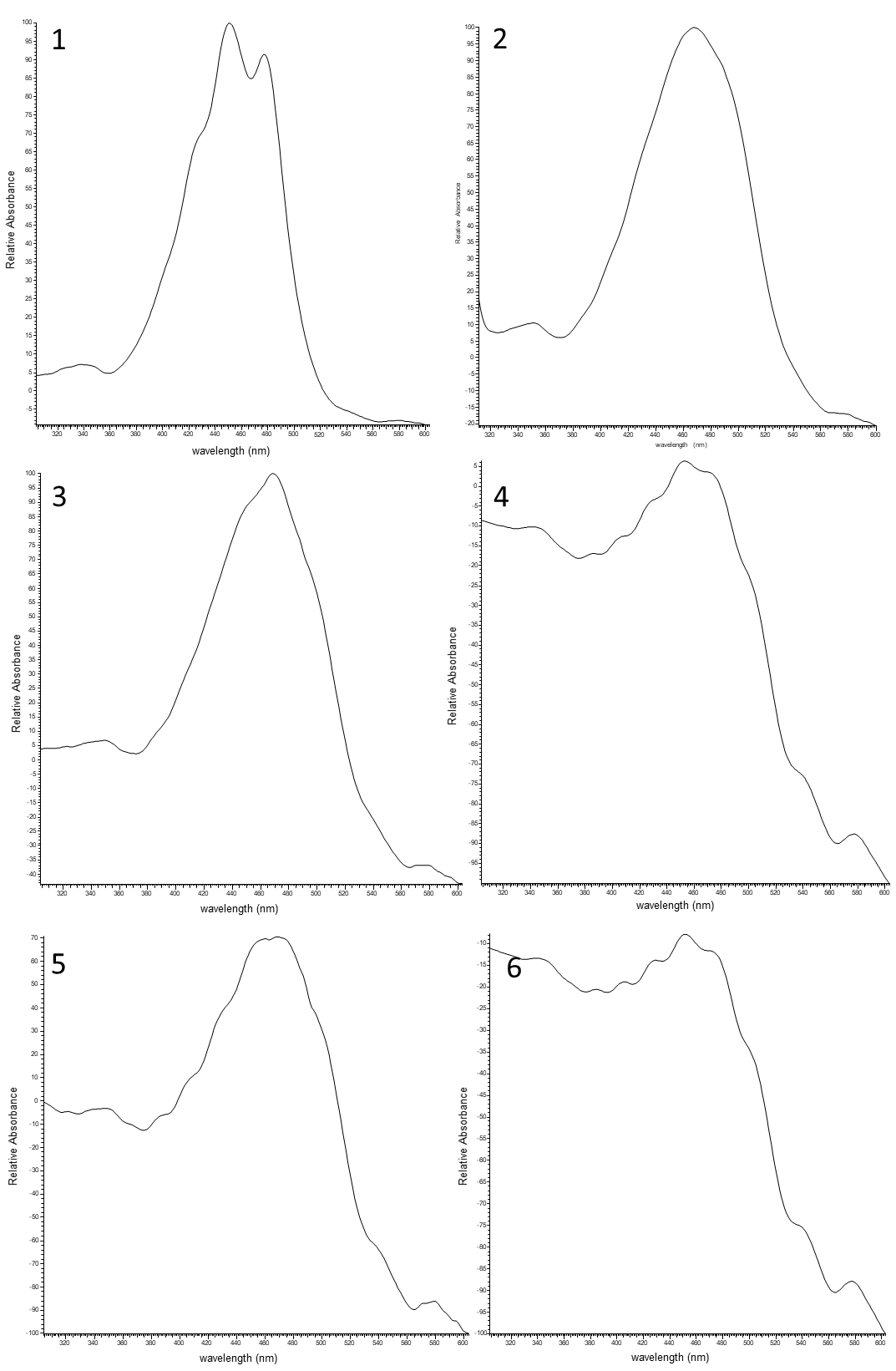


Supplementary Figure 7 UV-spectra of peak 1-6 (Supplementary Figure 6). UV-spectra of peak 7 was low in resolution due to low compound concentration.


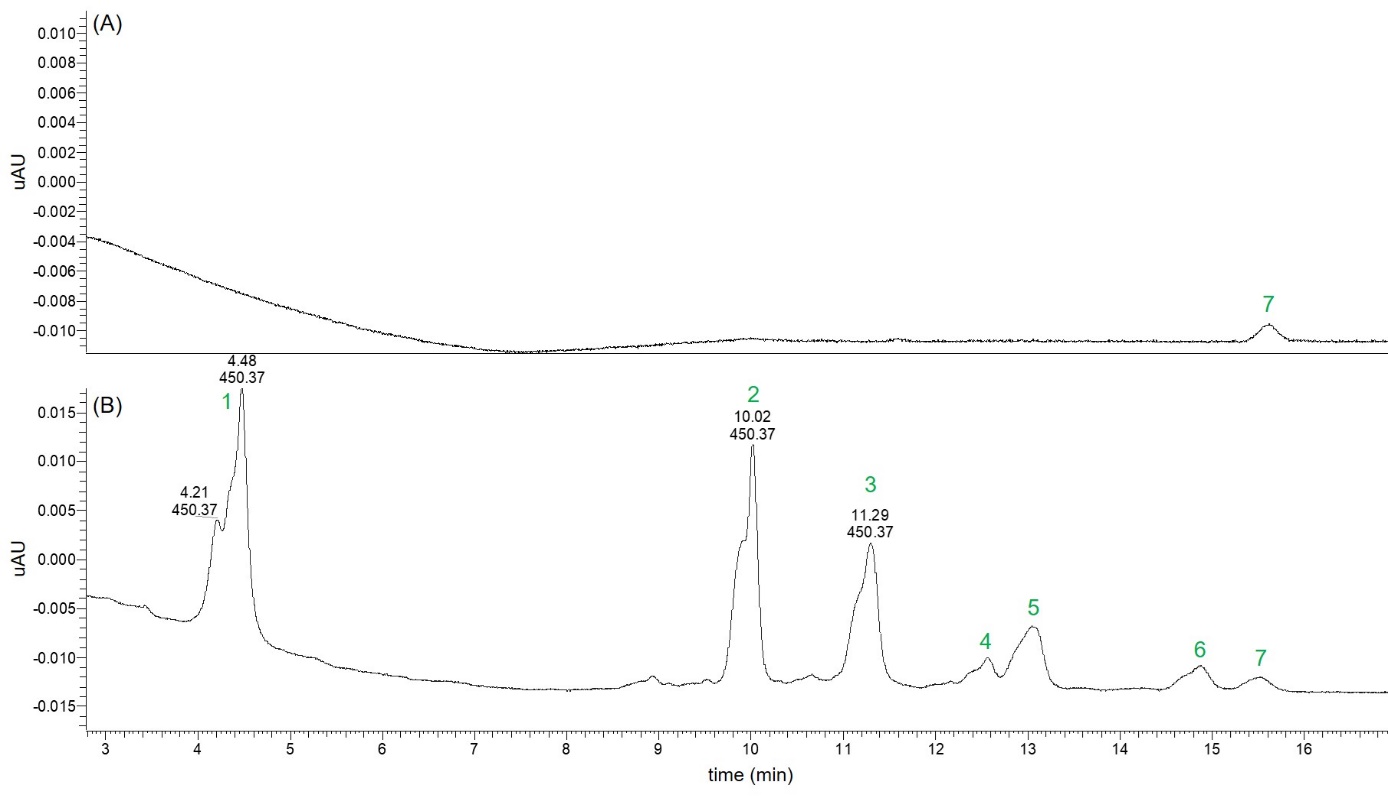


**Supplementary Figure 8** HPLC-DAD chromatograms (450 nm) of the methanol extracts of *S. capitis* subsp. *capitis* strain D2^T^ (A) compared to strain K1 (B).


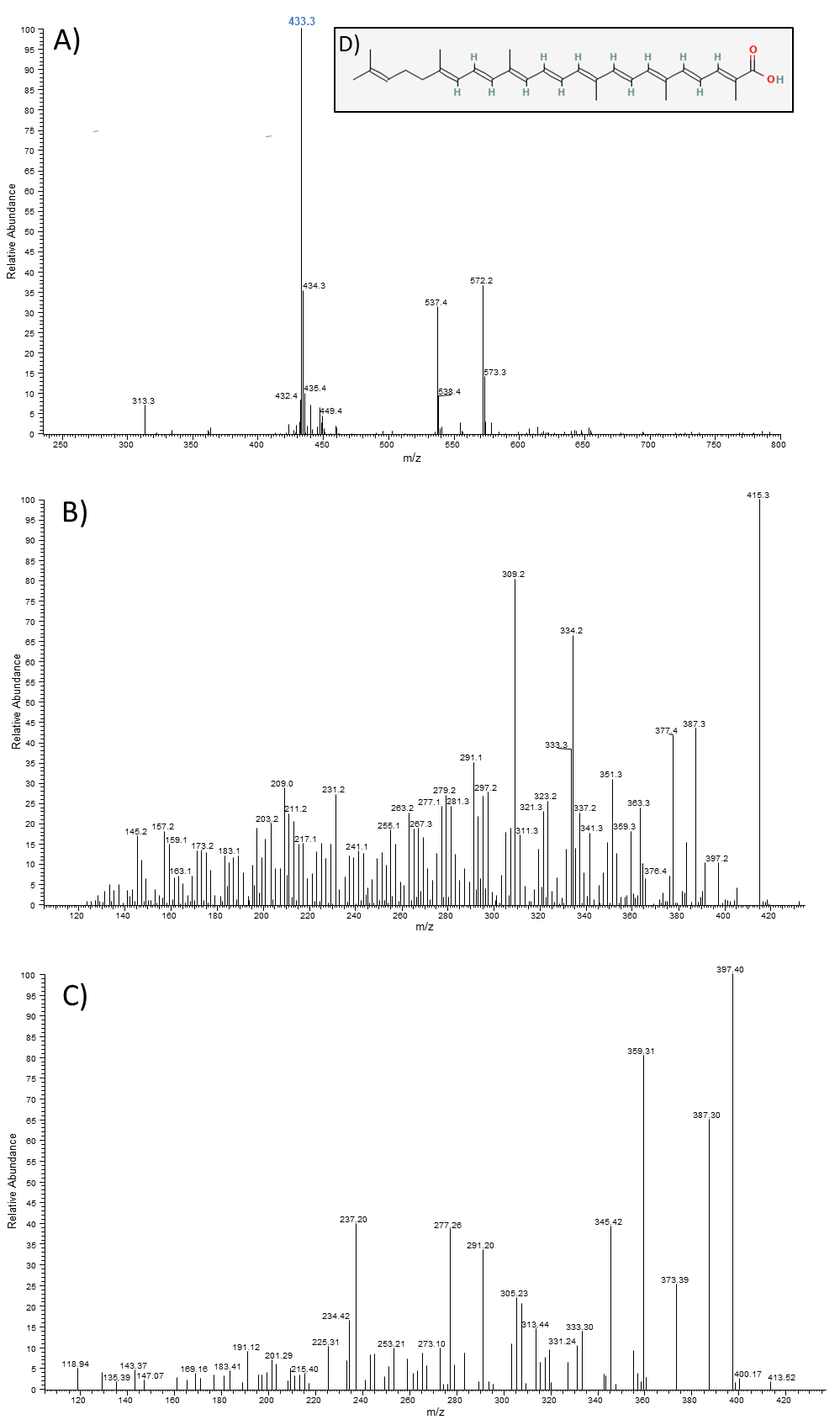


Supplementary Figure 9 MS spectra ([M+H]^+^) of tentatively identified all-*trans*-4,4'- diaponeurosporenoic acid. A) Full Scan. B) MS^2^ spectrum. C) MS^3^ spectrum (433🡪415). D) Chemical structure of 4,4'-diaponeurosporenoic acid derived from PubChem.


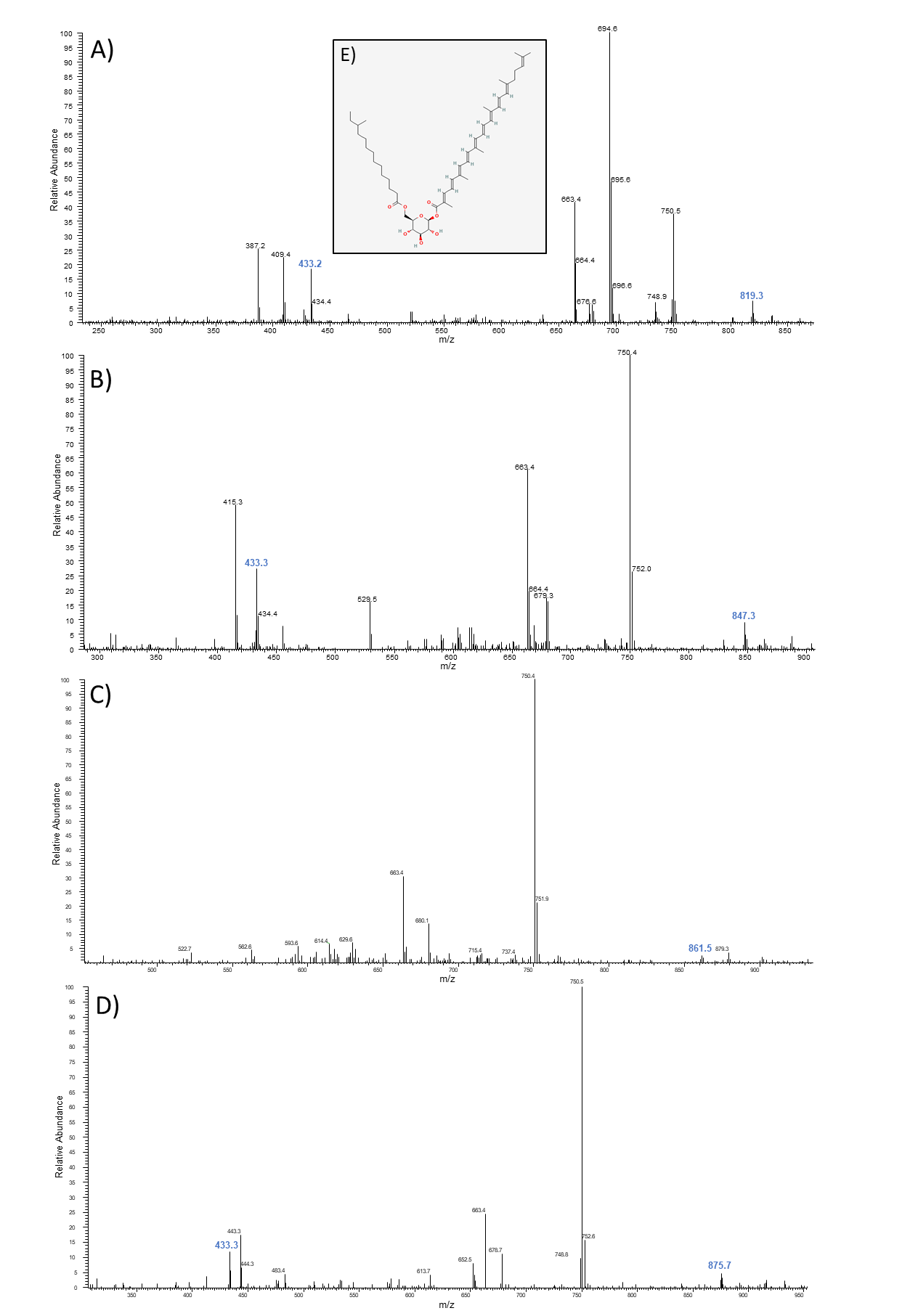


Supplementary Figure 10 Full MS scan ([M+H]^+^) of staphyloxanthin (A: *m/z* 819) and stapyhloxanthin-like compounds (B: *m/z* 847, C: *m/z* 861, D *m/z* 875). E) Chemical structure of staphyloxanthin derived from PubChem.

# Scavenging of reactive oxygen species (DPPH)


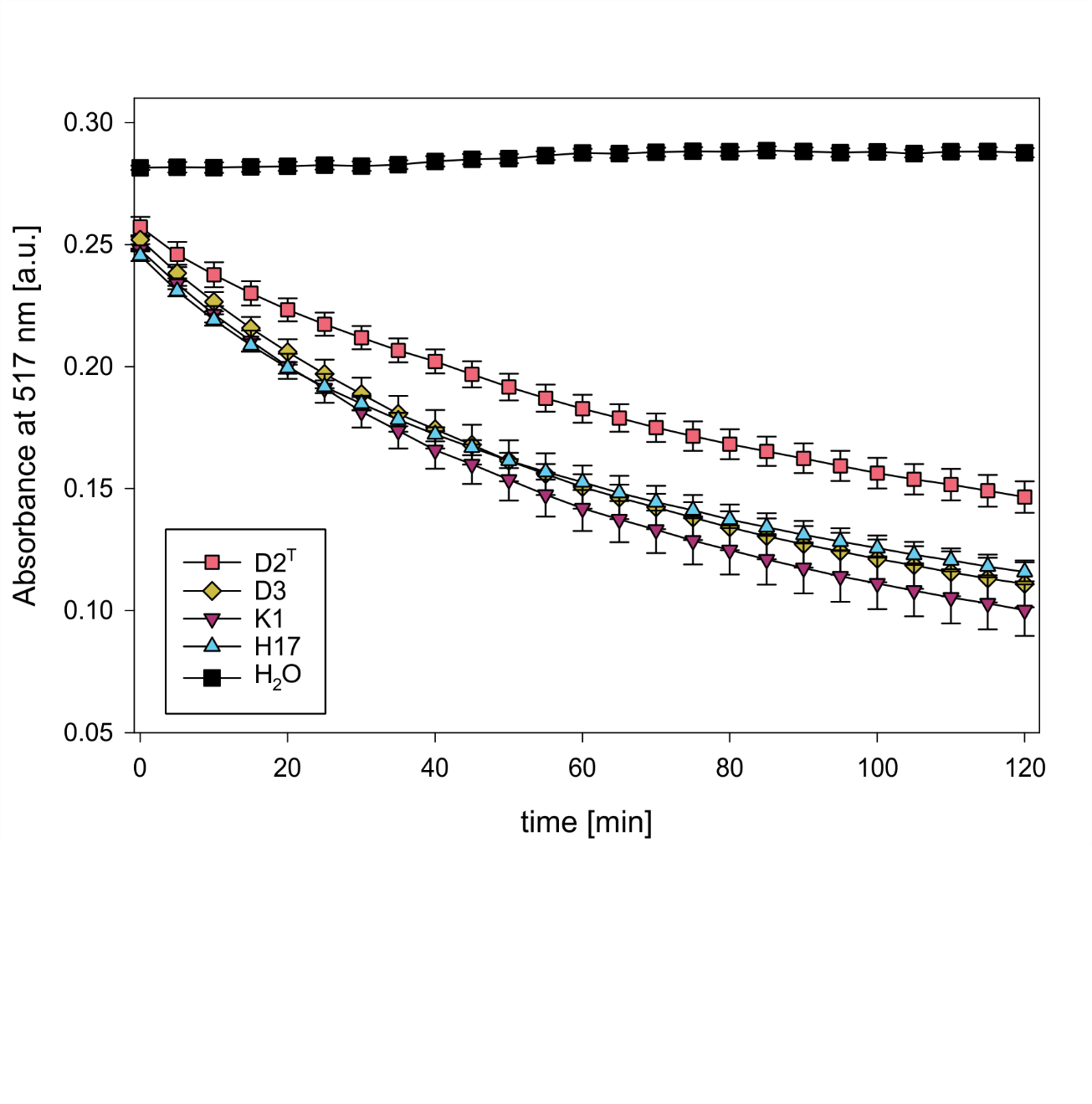


**Supplementary Figure 11** Average absorbance at 517 nm during incubation of methanol extracts of D2^T^, D3, H17 and K1 with DPPH. Negative control includes DPPH with water. Experiment was performed in triplicates. Error bars represent standard error.

# Survival in temperature stress


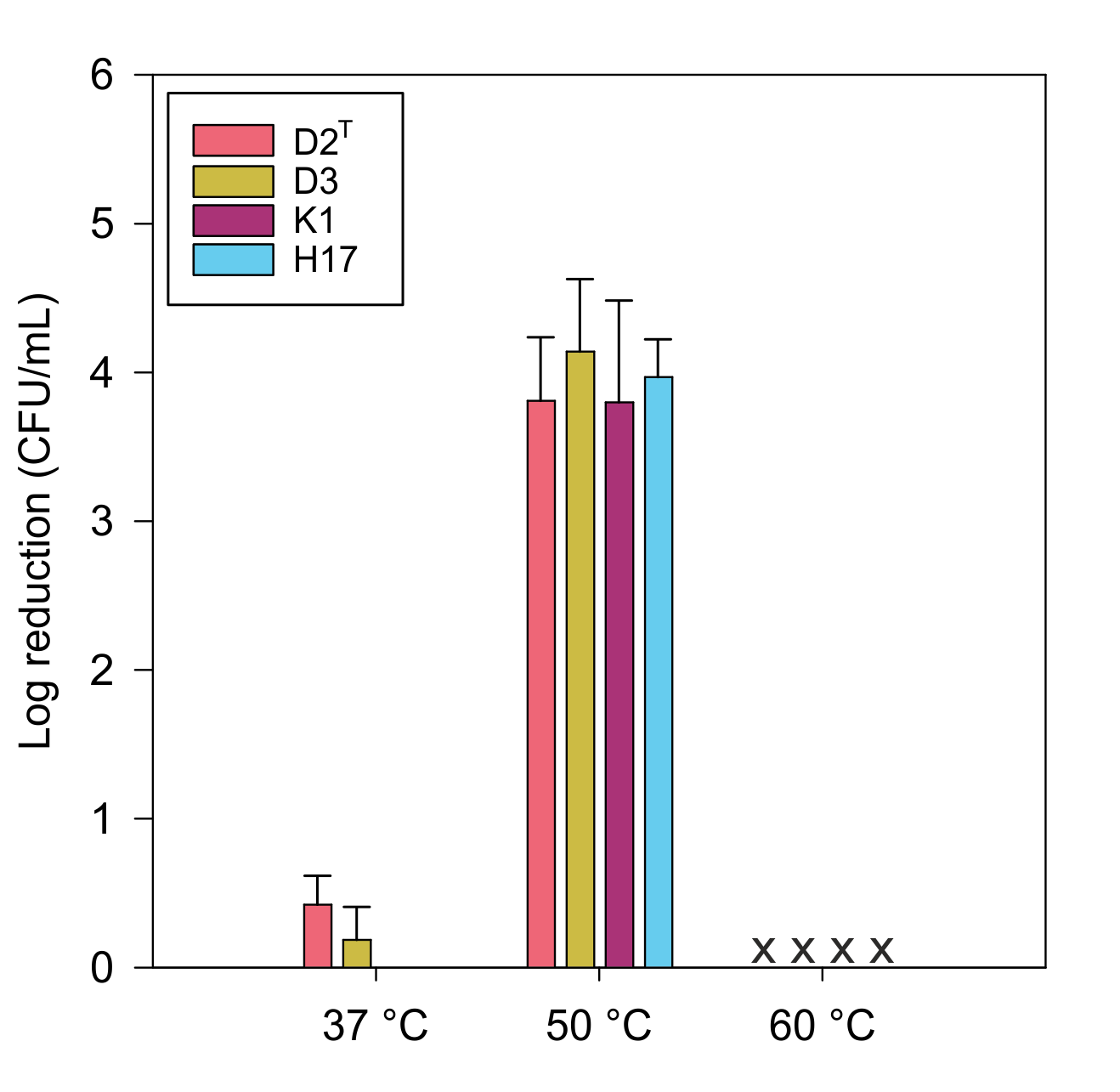


**Supplementary Figure 12** Log reduction of CFU/mL of *S. capitis* subsp. *capitis* strains D2^T^ (red), D3 (yellow), K1 (violet), H17 (blue) after 1h incubation at 37 °C, 50 °C and 60 °C. Experiment was performed in biological triplicates. Error bars represent the calculated standard error.

Supplementary Table 3 Overview of *crt* operon genes, gene products and localization in genomes of *S. capitis* subsp. *capitis* strains D2^T^, D3, K1 and H17. Assemblies are available in the Genbank database with the BioProject accession number PRJNA849394.

| **Contig** | **Start** | **Stop** | **Strand** | **Gene** | **Product** | **Reference** |
| --- | --- | --- | --- | --- | --- | --- |
| **D2^T^** | | | | | | |
| contig_1 | 344607 | 345092 | + | *crtO* | glycosyl-4,4'-diaponeurosporenoate acyltransferase | RefSeq:WP_002454050.1, SO:0001217, UniParc:UPI0001EF4E85, UniRef:UniRef100_A0A4U9T5Z4, UniRef:UniRef50_A0A654C2T6, UniRef:UniRef90_A0A1J4E794 |
| contig_1 | 345073 | 346584 | + | *crtP* | diapolycopene oxygenase | EC:1.14.99.44, KEGG:K10210, RefSeq:WP_002454049.1, SO:0001217, UniParc:UPI0001EF4E84, UniRef:UniRef100_A0A4U9T2G4, UniRef:UniRef50_Q99R73, UniRef:UniRef90_A0A4U9T2G4 |
| contig_1 | 346591 | 347703 | + | *crtQ* | 4,4'-diaponeurosporenoate glycosyltransferase | RefSeq:WP_103175481.1, SO:0001217, UniParc:UPI000CD0BFDC, UniRef:UniRef100_A0A4V6KIY9, UniRef:UniRef50_A0A654C1U8, UniRef:UniRef90_A0A4V6KIY9 |
| contig_1 | 347741 | 348604 | + | *crtM* | dehydrosqualene synthase | RefSeq:WP_002454047.1, SO:0001217, UniParc:UPI0001EF4E82, UniRef:UniRef100_A0A4U9TCN4, UniRef:UniRef50_A9JQL9, UniRef:UniRef90_A0A4U9TCN4 |
| contig_1 | 348617 | 348847 | + | *crtN* | Dehydrosqualene desaturase | SO:0001217, UniParc:UPI0001EF4E81, UniRef:UniRef100_A0A4U9TB88, UniRef:UniRef50_A0A4U9TB88, UniRef:UniRef90_A0A4U9TB88 |
| contig_1 | 348850 | 349974 | + | *crtN* | Dehydrosqualene desaturase | SO:0001217, UniParc:UPI0002FCF964, UniRef:UniRef100_A0A4U9T4Z8, UniRef:UniRef50_A0A4U9T4Z8, UniRef:UniRef90_A0A4U9T4Z8 |
| **D3** | | | | | | |
| contig_1 | 386093 | 386578 | + | *crtO* | glycosyl-4,4'-diaponeurosporenoate acyltransferase | RefSeq:WP_049427843.1, SO:0001217, UniParc:UPI00066AA718, UniRef:UniRef100_A0A1J4E794, UniRef:UniRef50_A0A654C2T6, UniRef:UniRef90_A0A1J4E794 |
| contig_1 | 386580 | 388070 | + | *crtP* | diapolycopene oxygenase | EC:1.14.99.44, KEGG:K10210, RefSeq:WP_030058724.1, SO:0001217, UniParc:UPI0004ABCF1F, UniRef:UniRef100_A0A848F1L8, UniRef:UniRef50_Q99R73, UniRef:UniRef90_A0A4U9T2G4 |
| contig_1 | 388077 | 389189 | + | *crtQ* | 4,4'-diaponeurosporenoate glycosyltransferase | RefSeq:WP_030058726.1, SO:0001217, UniParc:UPI0004AACC43, UniRef:UniRef100_A0A7Z7YVS7, UniRef:UniRef50_A0A654C1U8, UniRef:UniRef90_A0A4V6KIY9 |
| contig_1 | 389227 | 390090 | + | *crtM* | dehydrosqualene synthase | RefSeq:WP_030058728.1, SO:0001217, UniParc:UPI0004A9FF1A, UniRef:UniRef100_A0A7Z7YW25, UniRef:UniRef50_A9JQL9, UniRef:UniRef90_A0A4U9TCN4 |
| contig_1 | 390103 | 391608 | + | *crtN* | dehydrosqualene desaturase | EC:1.3.8.2, KEGG:K10209, RefSeq:WP_049427845.1, SO:0001217, UniParc:UPI00066AD76D, UniRef:UniRef100_UPI00066AD76D, UniRef:UniRef50_Q4L975, UniRef:UniRef90_A0A7X9ZJM6 |
|  |  |  |  |  |  |  |
| **K1** | | | | | | |
| contig_1 | 2099965 | 2101470 | - | *crtN* | dehydrosqualene desaturase | EC:1.3.8.2, KEGG:K10209, RefSeq:WP_023351099.1, SO:0001217, UniParc:UPI0003BF5B55, UniRef:UniRef100_UPI0003BF5B55, UniRef:UniRef50_Q4L975, UniRef:UniRef90_A0A7X9ZJM6 |
| contig_1 | 2101483 | 2102346 | - | *crtM* | dehydrosqualene synthase | RefSeq:WP_023351098.1, SO:0001217, UniParc:UPI0003BE0CA6, UniRef:UniRef100_UPI00066C6017, UniRef:UniRef50_A9JQL9, UniRef:UniRef90_A0A4U9TCN4 |
| contig_1 | 2102384 | 2103496 | - | *crtQ* | 4,4'-diaponeurosporenoate glycosyltransferase | RefSeq:WP_023351097.1, SO:0001217, UniParc:UPI0003BE2CF9, UniRef:UniRef100_UPI0003BE2CF9, UniRef:UniRef50_A0A654C1U8, UniRef:UniRef90_A0A4V6KIY9 |
| contig_1 | 2103503 | 2104993 | - | *crtP* | diapolycopene oxygenase | EC:1.14.99.44, KEGG:K10210, RefSeq:WP_049326541.1, SO:0001217, UniParc:UPI00066DADC4, UniRef:UniRef100_UPI00066DADC4, UniRef:UniRef50_Q99R73, UniRef:UniRef90_A0A4U9T2G4 |
| contig_1 | 2104995 | 2105480 | - | *crtO* | glycosyl-4,4'-diaponeurosporenoate acyltransferase | RefSeq:WP_023351095.1, SO:0001217, UniParc:UPI0003BE68DF, UniRef:UniRef100_UPI001D124E81, UniRef:UniRef50_A0A654C2T6, UniRef:UniRef90_A0A1J4E794 |
| **H17** | | | | | | |
| contig_1 | 398169 | 398654 | + | *crtO* | glycosyl-4,4'-diaponeurosporenoate acyltransferase | RefSeq:WP_023351095.1, SO:0001217, UniParc:UPI0003BE68DF, UniRef:UniRef100_UPI001D124E81, UniRef:UniRef50_A0A654C2T6, UniRef:UniRef90_A0A1J4E794 |
| contig_1 | 398656 | 400146 | + | *crtP* | diapolycopene oxygenase | EC:1.14.99.44, KEGG:K10210, RefSeq:WP_049326541.1, SO:0001217, UniParc:UPI00066DADC4, UniRef:UniRef100_UPI00066DADC4, UniRef:UniRef50_Q99R73, UniRef:UniRef90_A0A4U9T2G4 |
| contig_1 | 400153 | 401265 | + | *crtQ* | 4,4'-diaponeurosporenoate glycosyltransferase | RefSeq:WP_023351097.1, SO:0001217, UniParc:UPI0003BE2CF9, UniRef:UniRef100_UPI0003BE2CF9, UniRef:UniRef50_A0A654C1U8, UniRef:UniRef90_A0A4V6KIY9 |
| contig_1 | 401303 | 402166 | + | *crtM* | dehydrosqualene synthase | RefSeq:WP_023351098.1, SO:0001217, UniParc:UPI0003BE0CA6, UniRef:UniRef100_UPI00066C6017, UniRef:UniRef50_A9JQL9, UniRef:UniRef90_A0A4U9TCN4 |
| contig_1 | 402179 | 403684 | + | *crtN* | dehydrosqualene desaturase | EC:1.3.8.2, KEGG:K10209, RefSeq:WP_023351099.1, SO:0001217, UniParc:UPI0003BF5B55, UniRef:UniRef100_UPI0003BF5B55, UniRef:UniRef50_Q4L975, UniRef:UniRef90_A0A7X9ZJM6 |

Supplementary Table 4 Multiple Sequence Alignment of amino acid sequences of *crt* operon of *S. capitis* subsp. *capitis* strains D2^T^, D3, K1 and H17 with MUSCLE (3.8).

| **Glycosyl-4,4'-diaponeurosporenoate acyltransferase (*crtO*)** | |
| --- | --- |
| D2^T^ | MKAIKSITRLIILHSLFWFVVQMSIAHLGTRIPKSFFEKYSYLFRSFQWEENGELWDHTL |
| D3 | MKAIKSITRLIILHSLFWFVVQMSIAHLGTRIPKSFFEKYSCLFRSFQWEENGELWDHTL |
| K1 | MKAIKSITRLIILHSLFWFVVQMSIAHIGTRIPKSFFEKYSYLFRSFQWEENGELWDHTL |
| H17 | MKAIKSITRLIILHSLFWFVVQMSIAHIGTRIPKSFFEKYSYLFRSFQWEENGELWDHTL |
|  | ***************************:************* ****************** |
| D2^T^ | KVSKWKRLIPEGAQINKDIYDKSKLSLNIEETRRLLLEMRRAELIHWISILPVWLFVKAP |
| D3 | KVSKWKRLIPEGAQINKDIYDKSKLSVNIEQTRRLLLEMRRAELIHWISILSVWLFVKAP |
| K1 | KVSKWKRLIPEGAQINKDIYDKSKLSVNIEETRRLLLEMRRAELIHWISILPVWLFVKAP |
| H17 | KVSKWKRLIPEGAQINKDIYDKSKLSVNIEETRRLLLEMRRAELIHWISILPVWLFVKAP |
|  | **************************:***:********************.******** |
| D2^T^ | RYIKMINIAYVLFSHLPVIVAQRYNRPRIERLIRIIEKRGK |
| D3 | RYIKMINIAYVTFSHLPVIVAQRYNRPRIERLIRIIEKRGK |
| K1 | RYIKMINIAYVLFSHLPVIVAQRYNRPRIERLIRIIEKRGK |
| H17 | RYIKMINIAYVLFSHLPVIVAQRYNRPRIERLIRIIEKRGK |
|  | *********** ***************************** |
| **Diapolycopene oxygenase (*crtP*)** | |
| D2^T^ | LKSEVSNITQSILVIGGGLGGISAAIRMAQSGYKVTLFEQNNHIGGKVNRLEEQGFGFDL |
| D3 | -------MTQSILVIGGGLGGISAAIRMAQSGYKVTLFEQNNHIGGKVNRLEKQGFSFDL |
| K1 | -------MTQSILVIGGGLGGISAAIRMAQSGYKVTLFEQNNHIGGKVNRLEKQGFGFDL |
| H17 | -------MTQSILVIGGGLGGISAAIRMAQSGYKVTLFEQNNHIGGKVNRLEKQGFGFDL |
|  | :********************************************:***.*** |
| D2^T^ | GPSILTMPYIFERLFNYSGKNMADYIEIERLPLQWRSFFPDGEIIDLYENTEATLLNNLQ |
| D3 | GPSILTMPYIFERLFNYSGKNMADYIEIERLPLQWRSFFPDGEIIDLYENTEATLLNNLQ |
| K1 | GPSILTMPYIFERLFNYSGKNMADYIEIERLPLQWRSFFPDGEIIDLYENTEATLLNNLQ |
| H17 | GPSILTMPYIFERLFNYSGKNMADYIEIERLPLQWRSFFPDGEIIDLYENTEATLLNNLQ |
|  | ************************************************************ |
| D2^T^ | LGQEDKKELDNYLKYTKRIHRFTEKGYFNFGLDTLREIIKYHGTFKALRGFDYFSTMQQA |
| D3 | LGQEDKKELDNYLKYTKRIHRFTEKGYFNFGLDTLREIIKYHGPFKALRGFDYFSTMQQA |
| K1 | LGQEDKKELDNYLKYTKRIHRFTEKGYFNFGLDTLREIIKYHGPFKALRGFDYFSTMQQA |
| H17 | LGQEDKKELDNYLKYTKRIHRFTEKGYFNFGLDTLREIIKYHGPFKALRGFDYFSTMQQA |
|  | *******************************************.**************** |
| D2^T^ | IHRYISNPKLRDMLGYFIKYVGSSSYDAPAVLSMLFHMQQEQGLWYVKGGIHKLALALKQ |
| D3 | IHRYISNPKLRDMLGYFIKYVGSSSYDAPAVLSMLFHMQQEQGLWYVKGGIHKLALALKQ |
| K1 | INRYISNPKLRDMLGYFIKYVGSSSYDAPAVLSMLFHMQQEQGLWYVKGGIHKLALALKQ |
| H17 | INRYISNPKLRDMLGYFIKYVGSSSYDAPAVLSMLFHMQQEQGLWYVKGGIHKLALALKQ |
|  | *:********************************************************** |
| D2^T^ | LAIEEGVDIQMGVAVENIKTYHQRVTSVRLSSGKYVEANYIISNMEVIPTYRELLHFNQQ |
| D3 | LAIEEGVDIQMGVAVENIKTYHQRVTGVRLSSGKYVEADYIISNMEVIPTYRELLHFNQQ |
| K1 | LAIEEGVDIQIRVAVENIKTYHQRVTGVRLSSGKYVEADYIISNMEVIPTYRELLHFNQQ |
| H17 | LAIEEGVDIQIRVAVENIKTYHQRVTGVRLSSGKYVEADYIISNMEVIPTYRELLHFNQQ |
|  | **********: **************.***********:********************* |
| D2^T^ | KIDQLESTYEPASSGYVLHLGVDKSYSQLAHHNFFFSKDSKKNYDEIFHQKVLPQDPTIY |
| D3 | KIDQLERTYEPASSGYVLHLGIDKSYSQLAHHNFFFSKDSKKNYDEVFHQKVLPQDPTIY |
| K1 | KIDQLERTYEPASSGYVLHLGIDKSYSQLAHHNFFFSKDSKKNYDEVFHQKVLPQDPTIY |
| H17 | KIDQLERTYEPASSGYVLHLGIDKSYSQLAHHNFFFSKDSKKNYDEVFHQKVLPQDPTIY |
|  | ****** **************:************************:************* |
| D2^T^ | LVNVNKTDEQQASQGYENIKVLPHIPYIQDKPFKKEEYAAFKERVLTKLERMGLTDLRAH |
| D3 | LVNVNKTDEQQAPQGYENIKVLPHIPYIQDKPFKKEEYAAFKERVLTKLERMGLTDLRAH |
| K1 | LVNVNKTDEQQAPQGYENIKVLPHIPYIQDKPFKKEEYAAFKERVLTKLERMGLTDLRAH |
| H17 | LVNVNKTDEQQAPQGYENIKVLPHIPYIQDKPFKKEEYAAFKERVLTKLERMGLTDLRAH |
|  | ************.*********************************************** |
| D2^T^ | IIFEDIWTPEDIRHNYRSNRGAIYGIVADKKKNKGFKFPKQSEYFDNLFFVGGSVNPGGG |
| D3 | IIYEDIWTPEDIQHNYRSNRGAIYGVVADKKKNKGFKFPKQSEYFDNLFFVGGSVNPGGG |
| K1 | IIYEDIWTPEDIQHNYRSNRGAIYGVVADKKKNKGFKFPKQSEYFDNLFFVGGSVNPGGG |
| H17 | IIYEDIWTPEDIQHNYRSNRGAIYGVVADKKKNKGFKFPKQSEYFDNLFFVGGSVNPGGG |
|  | **:*********.************:********************************** |
| D2^T^ | MPMVTLSGMQVADKINAIEAGRS |
| D3 | MPMVTLSGMQVADKINAIEAGRS |
| K1 | MPMVTLSGMQVADKINAIEAGRS |
| H17 | MPMVTLSGMQVADKINAIEAGRS |
|  | *********************** |
| **4,4'-Diaponeurosporenoate glycosyltransferase (*crtQ*)** | |
| D2^T^ | LKLIQLLLSLASVISLICGKFIYNRRHTLSRSKHSTIHSNELTVIIPARDEAQRLPNLLR |
| D3 | LKLIQLLLSLASVISLICGKFIYNRRHTLSRSKHSTIHSNELTVIIPARDEAQRLPHLLR |
| K1 | LKLIQLLLSLASVISLICGKLIYNRRHALSRSKHSTIHSNELTVIIPARDEAQRLPNLLR |
| H17 | LKLIQLLLSLASVISLICGKLIYNRRHALSRSKHSTIHSNELTVIIPARDEAQRLPNLLR |
|  | ********************:******:****************************:*** |
| D2T | SLTKQRGIYEIIVMDDASQDGTSEVAKAYGATVYETKEDSQWYDKSHACYQGAQHVQTPL |
| D3 | SLTKQHGIYEIIVMDDASQDGTSEVAKAYGATVYETKEDSQWYGKSHACYQGAQHVQTPL |
| K1 | SLTKQRGIYEIIVMDDASQDGTSEVAKAYGATVYETKEDSQWYGKSHACYQGAQHVQTPL |
| H17 | SLTKQRGIYEIIVMDDASQDGTSEVAKAYGATVYETKEDSQWYGKSHACYQGAQHVQTPL |
|  | *****.*************************************.**************** |
| D2^T^ | MMFVDADVIFNSHAIEAILNSFAQQGNQGLLSIQPYHETFKFYESLSAIFNLMTIVGMNR |
| D3 | MMFVDADVIFNSHAIEAILNSFAKQGNQGLLSIQPYHETFKFYESLSAIFNLMTIVGMNR |
| K1 | MMFVDADVIFNSHAIEAILNSFAQQGNQGLLSIQPYHETFKFYESLSAIFNLMTIVGMNR |
| H17 | MMFVDADVIFNSHAIEAILNSFAQQGNQGLLSIQPYHETFKFYESLSAIFNLMTIVGMNR |
|  | ***********************:************************************ |
| D2^T^ | FSSLASKSNDYTAFGPVTIMNKEDYFKTGGHKNARNTIIEGFALGEAFSQSNLPITVYEG |
| D3 | FSSLAGKFNNYTAFGPVTIMNKEDYFKTGGHKNARNTIIEGFALGEAFSQSNLPVTVYEG |
| K1 | FSSLAGKFNNYTAFGPVTIMNKEDYFKTGGHKNARNTIIEGFALGEAFSQSNLPVTVYEG |
| H17 | FSSLAGKFNNYTAFGPVTIMNKEDYFKTGGHKNARNTIIEGFALGEAFSQSNLPVTVYEG |
|  | *****.* *:********************************************:***** |
| D2^T^ | SEYVKFRMYEEGLRSLIQGWTKHFSVGANQTEPQVMLAIIMWLMGSLTSTMALLLGWIAK |
| D3 | SEYVKFRMYEEGLRSLIQGWTKHFSVGANQTEPKVMLAIVMWLMGSLTSTMALLLGWIAK |
| K1 | SEYVKFRMYEEGLRSLIQGWTKHFSVGANQTEPKVMLAIVMWLMGSLTSTMALLLGWIAK |
| H17 | SEYVKFRMYEEGLRSLIQGWTKHFSVGANQTEPKVMLAIVMWLMGSLTSTMALLLGWIAK |
|  | *********************************:*****:******************** |
| D2^T^ | PISLIFSGIVYILYTWEFVSLHRRVGAFSIILLILHPILFVFFIIIFINSWRHAHFSKKV |
| D3 | PISLIFSGIVYILYTWEFVSLHRRVGAFSIILLILHPILFVFFIIIFINSWRHAHFSKKV |
| K1 | PISLIFSGIVYILYTWEFVSLHRRVGAFSIILLILHPILFVFFIIIFINSWRHAHFSKKV |
| H17 | PISLIFSGIVYILYTWEFVSLHRRVGAFSIILLILHPILFVFFIIIFINSWRHAHFSKKV |
|  | ************************************************************ |
| D2^T^ | KWKGRTFDIS |
| D3 | KWKGRTFDIS |
| K1 | KWKSRTFDIS |
| H17 | KWKSRTFDIS |
|  | ***.****** |
| **Dehydrosqualene synthase (*crtM*)** | |
| D2^T^ | MTSNEKNFKYCHQIMKEHSKSFSYAFDYLPENERKAVWAIYAVCRIIDDSIDVHENPQIL |
| D3 | MTSNEKNFKYCHQIMKEHSKSFSYAFDYLPENERKAVWAIYAVCRIIDDSIDVHENPQIL |
| K1 | MTSNEKNFKYCHQIMKEHSKSFSYAFDYLPENERKAVWAIYAVCRIIDDSIDVHENPQIL |
| H17 | MTSNEKNFKYCHQIMKEHSKSFSYAFDYLPENERKAVWAIYAVCRIIDDSIDVHENPQIL |
|  | ************************************************************ |
| D2^T^ | KNIHDDIITIEKNHDVENYEFKSNQMIMEALYTVSQNFTIEYQSFYNLIQTVYEDQDFEM |
| D3 | KNIHEDIITIEKNHDVENYEFKSNQMIMEALYTVSQNFTIEYQSFYNLIQTVYEDQDFEM |
| K1 | KNIHDDIITIEKNHDVENYEFKSNQMIMEALYTVSQNFTIEYQSFYNLIQTVYEDQEFEM |
| H17 | KNIHDDIITIEKNHDVENYEFKSNQMIMEALYTVSQNFTIEYQSFYNLIQTVYEDQEFEM |
|  | ****:***************************************************:*** |
| D2^T^ | FETDDELLNYCYGVAGTVGEVLTPVLAEQPNEETYRIARKLGEALQITNILRDVGEDFEN |
| D3 | FETDDELLNYCYGVAGTVGEVLTPVLAEQPNEETYRIARKLGEALQITNILRDVGEDFEN |
| K1 | FETDDELLNYCYGVAGTVGEVLTPVLAEQPNEETYRIARKLGEALQITNILRDVGEDFEN |
| H17 | FETDDELLNYCYGVAGTVGEVLTPVLAEQPNEETYRIARKLGEALQITNILRDVGEDFEN |
|  | ************************************************************ |
| D2^T^ | DRIYFSKSSLSRFDVSIENDFNNGVSQQYIDLWEHHAKLAQEDYDIALSNLGVFNKEAQP |
| D3 | DRIYFSKSSLSRFDVSIENNFNNGVSQQYIDLWEHHAKLAQEDYDIALSNLGVFNKEAQP |
| K1 | DRIYFSKSSLSRFDVSIENNFNNGVSQQYIDLWEHHAKLAQEDYDIALSNLGVFNKEAQP |
| H17 | DRIYFSKSSLSRFDVSIENNFNNGVSQQYIDLWEHHAKLAQEDYDIALSNLGVFNKEAQP |
|  | *******************:**************************************** |
| D2^T^ | IIELASIIYRGILDEVRKASYTLHRRVYVSKLDKVKMYKTIKKKYHL |
| D3 | IIELASIIYRGILDEVRKASYTLHRRVYVSKLDKVKMYKTIKKKYHL |
| K1 | IIELASIIYRGILDEVRKASYTLHRRVYVSKLDKVKMYKIIKKKYHL |
| H17 | IIELASIIYRGILDEVRKASYTLHRRVYVSKLDKVKMYKIIKKKYHL |
|  | *************************************** ******* |
| **Dehydrosqualene desaturase (*crtN*)** | |
| D2^T^ | MNIAVIGAGVTGLAAAARLASQGNNVTIFEKNGYPGGRMSQFTKDGFTFDKGPSIVMIPRYTRQYLKKAVRNLKTT |
| **Dehydrosqualene desaturase (*crtN*)** | |
| D2^T^ | ------------------------------------------------------------ |
| D3 | MNIAVIGAGVTGLAAAARLASQGNNVTIFEKNGYPGGRMSQFTKDGFTFDKGPSIVMIPE |
| K1 | MNIAVIGAGVTGLAAAARLASQGNNVTIFEKNGYPGGRMSQFTKDGFTFDKGPSIVMIPE |
| H17 | MNIAVIGAGVTGLAAAARLASQGNNVTIFEKNGYPGGRMSQFTKDGFTFDKGPSIVMIPE |
|  |  |
| D2^T^ | ------------------MEQLRYIYDVYFEKNDKVRVPTDLAELQETLESIEPGTTHGF |
| D3 | VYKAVFEESGKKFEDYVDMEQLRYIYDVYFGKNDKVRVPTDLAELQETLESIEPGTTHGF |
| K1 | VYKAVFEESGKKFEDYVDMEQLRYIYDVYFGKNDKVRVPTDLAELQETLESIEPGTTHGF |
| H17 | VYKAVFEESGKKFEDYVDMEQLRYIYDVYFGKNDKVRVPTDLAELQETLESIEPGTTHGF |
|  | ************ ***************************** |
| D2^T^ | MSFLTDVYKRYEIARYHFLEKTYRKVTDFYNVDSLIKGLKLKTLNNADNLIENYIDNERI |
| D3 | MSFLTDVYKRYEIARYHFLEKTYRKVTDFYNVDSLIKGLKLKTLNNADNLIENYIDNERI |
| K1 | MSFLTDVYKRYEIARYHFLEKTYRKVTDFYNVDSLIKGLKLKTLNNADNLIENYIDNERI |
| H17 | MSFLTDVYKRYEIARYHFLEKTYRKVTDFYNVDSLIKGLKLKTLNNADNLIENYIDNERI |
|  | ************************************************************ |
| D2^T^ | QKLLAFQMLYIGIDPKRGPSLYSIIPMVEMMFGVHFIKGGMYGLTRGLVKLNKDLDVNIQ |
| D3 | QKLLAFQMLYIGIDPKRGPSLYSIIPMVEMMFGVHFIKGGMYGLTRGLVKLNKDLDVNIQ |
| K1 | QKLLAFQMLYIGIDPKRGPSLYSIIPMVEMMFGVHFIKGGMYGLTRGLVKLNKDLDVNIQ |
| H17 | QKLLAFQMLYIGIDPKRGPSLYSIIPMVEMMFGVHFIKGGMYGLTRGLVKLNKDLDVNIQ |
|  | ************************************************************ |
| D2^T^ | LNSNIEEIIIDPKYKRADGVRVNGLVQRFDKVLCTADFPYAAEKLMPSHSPVKKYKPSKI |
| D3 | LNSNIEEIIIDPKYKRADGVRVNGLVQRFDKVLCTADFPYAAEKLMPSHSPVKKYKPSKI |
| K1 | LNSNIEEIIIDPKYKRADGIRVNDLVQRFDKVLCTADFPYAAEKLMPSHSPVKKYKPSKI |
| H17 | LNSNIEEIIIDPKYKRADGIRVNDLVQRFDKVLCTADFPYAAEKLMPSHSPVKKYKPSKI |
|  | *******************:***.************************************ |
| D2^T^ | EKLDYSCSAFLMYVGIDKDVTSEMLLHNVIFSQQFRQNIGEIFNGSLSEDPSIYVYVPAV |
| D3 | EKLDYSCSAFLMYIGIDKDVTSEMLLHNVIFSQQFRQNIDEIFNGSLSEDPSIYVYVPAV |
| K1 | EKLDYSCSAFLMYIGIDKDVTSEMLLHNVIFSQQFRQNIDEIFNGSLSEDPSIYVYVPAV |
| H17 | EKLDYSCSAFLMYIGIDKDVTSEMLLHNVIFSQQFRQNIDEIFNGSLSEDPSIYVYVPAV |
|  | *************:*************************.******************** |
| D2^T^ | GDRSMAPEGQTGIYVLMPTPELKTGHIDWKDENVIKKVKDIIYRQLETIDVLEDVKSNVI |
| D3 | GDRSMAPEGQTGIYVLMPTPELKTGHINWKDENVIRKVKDIIYRQLETIDVLEDVKSNVI |
| K1 | GDRSMAPEGQTGIYVLMPTPELKTGHINWKDENVIKKVKDIIYRQLETIDVLEDVKSHVI |
| H17 | GDRSMAPEGQTGIYVLMPTPELKTGHINWKDENVIKKVKDIIYRQLETIDVLEDVKSHVI |
|  | ***************************:*******.*********************:** |
| D2^T^ | SETVYTPIDFENDYNAKFGTAFGLMPTLAQSN---------------------------- |
| D3 | SETVYTPIDFENDYNAKFGTAFGLMPTLAQSNYYRPPNVSRDYKDLYFAGASTHPCAGVP |
| K1 | SETVYTPIDFENDYNAKFGTAFGLMPTLAQSNYYRPPNVSRDYKDLYFAGASTHPGAGVP |
| H17 | SETVYTPIDFENDYNAKFGTAFGLMPTLAQSNYYRPPNVSRDYKDLYFAGASTHPGAGVP |
|  | ******************************** |
| D2^T^ | --------------------- |
| D3 | IVLTSAKITVNEMLKDIDNNI |
| K1 | IVLTSAKITVNEMLKDIDNNI |
| H17 | IVLTSAKITVNEMLKDIDNNI |
|  |  |

Supplementary Table 5 Multiple Sequence Alignment of nucleic acid sequences of *crtN* of *S. capitis* subsp. *capitis* strains D2^T^, D3, K1 and H17 with CLUSTAL O (1.2.4).

| D2^T^ | ATGAATATTGCTGTAATAGGAGCAGGCGTCACTGGATTAGCAGCGGCAGCAAGACTTGCT |
| --- | --- |
| D3 | ATGAATATTGCTGTAATAGGAGCAGGCGTCACTGGATTAGCAGCGGCAGCAAGACTTGCT |
| K1 | ATGAATATTGCTGTAATAGGAGCAGGCGTCACTGGATTAGCAGCGGCAGCAAGACTTGCT |
| H17 | ATGAATATTGCTGTAATAGGAGCAGGCGTCACTGGATTAGCAGCGGCAGCAAGACTTGCT |
|  | ************************************************************ |
| D2^T^ | TCGCAAGGTAATAATGTAACAATTTTTGAAAAAAATGGGTATCCAGGTGGTCGAATGAGT |
| D3 | TCGCAAGGTAATAATGTAACAATTTTTGAAAAAAATGGGTATCCAGGTGGTCGAATGAGT |
| K1 | TCGCAAGGTAATAATGTAACAATTTTTGAAAAAAATGGGTATCCAGGTGGTCGAATGAGT |
| H17 | TCGCAAGGTAATAATGTAACAATTTTTGAAAAAAATGGGTATCCAGGTGGTCGAATGAGT |
|  | ************************************************************ |
| D2^T^ | CAATTCACAAAAGATGGCTTTACCTTTGACAAAGGTCCTTCTATCGTGATGATC-CCGAG |
| D3 | CAATTCACTAAAGATGGCTTTACCTTTGATAAAGGTCCTTCTATCGTGATGATTCCCGAG |
| K1 | CAATTCACTAAAGATGGCTTTACCTTTGATAAAGGTCCTTCTATCGTGATGATTCCCGAG |
| H17 | CAATTCACTAAAGATGGCTTTACCTTTGATAAAGGTCCTTCTATCGTGATGATTCCCGAG |
|  | ******** ******************** *********************** ***** |
| D2^T^ | GTATACAAGGCAGTATTTGAAGAAAGCGGTAAGAAATTTGAAGACTACGTAGA--TGGAA |
| D3 | GTATACAAGGCAGTATTTGAAGAAAGCGGTAAGAAATTTGAAGACTACGTAGATATGGAA |
| K1 | GTATACAAGGCAGTATTTGAAGAAAGCGGTAAGAAATTTGAAGACTACGTAGATATGGAA |
| H17 | GTATACAAGGCAGTATTTGAAGAAAGCGGTAAGAAATTTGAAGACTACGTAGATATGGAA |
|  | ***************************************************** ***** |
| D2^T^ | CAATTGCGCTATATTTATGATGTTTACTTTGAGAAAAATGACAAAGTAAGAGTACCTACT |
| D3 | CAATTGCGCTATATTTATGATGTTTACTTTGGGAAAAATGACAAAGTAAGAGTACCTACT |
| K1 | CAATTGCGCTATATTTATGATGTTTACTTTGGGAAAAATGACAAAGTAAGAGTACCTACT |
| H17 | CAATTGCGCTATATTTATGATGTTTACTTTGGGAAAAATGACAAAGTAAGAGTACCTACT |
|  | ******************************* **************************** |
| D2^T^ | GATTTAGCCGAACTTCAAGAAACGCTTGAAAGTATTGAACCAGGTACGACACATGGGTTT |
| D3 | GATTTAGCCGAACTTCAAGAAACGCTTGAAAGTATTGAACCAGGTACGACACATGGGTTT |
| K1 | GATTTAGCCGAACTTCAAGAAACGCTTGAAAGTATTGAACCAGGTACGACACATGGGTTT |
| H17 | GATTTAGCCGAACTTCAAGAAACGCTTGAAAGTATTGAACCAGGTACGACACATGGGTTT |
|  | ************************************************************ |
| D2^T^ | ATGTCATTTTTAACTGATGTTTACAAGCGATATGAAATTGCGCGCTATCATTTTTTAGAG |
| D3 | ATGTCATTTTTAACTGATGTTTACAAGCGATATGAAATTGCGCGCTATCATTTTTTAGAG |
| K1 | ATGTCATTTTTAACTGATGTTTACAAGCGATATGAAATTGCGCGCTATCATTTTTTAGAG |
| H17 | ATGTCATTTTTAACTGATGTTTACAAGCGATATGAAATTGCGCGCTATCATTTTTTAGAG |
|  | ************************************************************ |
| D2^T^ | AAAACATATAGAAAGGTTACGGATTTCTATAATGTAGATTCGCTTATTAAAGGTTTGAAA |
| D3 | AAAACATATAGAAAGGTTACGGATTTCTATAATGTAGATTCGCTTATTAAAGGTTTGAAA |
| K1 | AAAACATATAGAAAGGTTACGGATTTCTATAATGTAGATTCGCTTATTAAAGGTTTGAAA |
| H17 | ************************************************************ |
|  | TTAAAAACATTAAACAATGCTGATAATTTAATAGAAAATTATATTGATAATGAACGAATA |
| D2^T^ | TTAAAAACATTAAACAATGCTGATAATTTAATAGAAAATTATATTGATAATGAACGAATA |
| D3 | TTAAAAACATTAAACAATGCTGATAATTTAATAGAAAATTATATTGATAATGAACGAATA |
| K1 | TTAAAAACATTAAACAATGCTGATAATTTAATAGAAAATTATATTGATAATGAACGAATA |
| H17 | ************************************************************ |
| D2^T^ | CAAAAATTATTAGCATTTCAAATGTTATATATAGGAATTGATCCAAAACGCGGCCCCTCT |
| D3 | CAAAAATTATTAGCATTTCAAATGTTATATATAGGAATTGATCCAAAACGCGGCCCCTCT |
| K1 | CAAAAATTATTAGCATTTCAAATGTTATATATAGGAATTGATCCAAAACGCGGCCCCTCT |
| H17 | CAAAAATTATTAGCATTTCAAATGTTATATATAGGAATTGATCCAAAACGCGGCCCCTCT |
|  | ************************************************************ |
| D2^T^ | TTATACTCAATTATTCCTATGGTAGAAATGATGTTTGGTGTTCACTTTATTAAGGGGGGC |
| D3 | TTATACTCAATTATTCCTATGGTAGAAATGATGTTTGGTGTTCACTTTATTAAGGGGGGC |
| K1 | CTATACTCAATTATTCCTATGGTAGAAATGATGTTTGGTGTTCACTTTATTAAGGGGGGC |
| H17 | CTATACTCAATTATTCCTATGGTAGAAATGATGTTTGGTGTTCACTTTATTAAGGGGGGC |
|  | *********************************************************** |
| D2^T^ | ATGTATGGATTAACACGTGGACTTGTTAAATTAAATAAAGATTTAGATGTGAATATTCAA |
| D3 | ATGTATGGATTAACACGTGGACTTGTTAAATTAAATAAAGATTTAGATGTGAATATTCAA |
| K1 | ATGTATGGATTAACACGAGGACTTGTTAAATTAAATAAAGATTTAGATGTGAATATTCAA |
| H17 | ATGTATGGATTAACACGAGGACTTGTTAAATTAAATAAAGATTTAGATGTGAATATTCAA |
|  | ***************** ****************************************** |
| D2^T^ | TTAAATTCTAATATAGAAGAAATCATCATCGACCCTAAATATAAGCGTGCTGATGGCGTA |
| D3 | TTAAATTCTAATATAGAAGAAATCATCATCGACCCTAAATATAAGCGTGCTGATGGCGTA |
| K1 | TTAAATTCTAATATAGAAGAAATCATCATCGACCCTAAATATAAGCGTGCTGATGGCATA |
| H17 | TTAAATTCTAATATAGAAGAAATCATCATCGACCCTAAATATAAGCGTGCTGATGGCATA |
|  | ********************************************************* ** |
| D2^T^ | AGGGTTAATGGTCTTGTGCAACGATTCGATAAAGTATTATGTACAGCAGATTTTCCTTAT |
| D3 | AGGGTTAATGGTCTTGTGCAACGATTTGATAAAGTATTATGTACAGCAGATTTTCCTTAT |
| K1 | AGGGTTAATGATCTTGTGCAACGATTCGATAAAGTATTATGTACAGCAGATTTTCCTTAT |
| H17 | AGGGTTAATGATCTTGTGCAACGATTCGATAAAGTATTATGTACAGCAGATTTTCCTTAT |
|  | ********** *************** ********************************* |
| D2^T^ | GCAGCGGAAAAATTGATGCCTTCACATTCACCCGTAAAAAAATATAAACCTAGTAAAATT |
| D3 | GCAGCGGAAAAATTGATGCCTTCACATTCACCCGTAAAAAAATATAAACCTAGTAAAATT |
| K1 | GCAGCGGAAAAATTGATGCCTTCACATTCACCCGTAAAAAAATATAAACCTAGTAAAATT |
| H17 | GCAGCGGAAAAATTGATGCCTTCACATTCACCCGTAAAAAAATATAAACCTAGTAAAATT |
|  | ************************************************************ |
| D2^T^ | GAAAAATTAGATTATTCTTGTTCTGCATTTTTAATGTATGTTGGTATTGATAAAGATGTG |
| D3 | GAAAAATTAGATTATTCTTGTTCTGCATTTTTAATGTATATTGGTATTGATAAAGATGTG |
| K1 | GAAAAATTAGATTATTCTTGTTCTGCATTTTTAATGTATATTGGTATTGATAAAGATGTG |
| H17 | GAAAAATTAGATTATTCTTGTTCTGCATTTTTAATGTATATTGGTATTGATAAAGATGTG |
|  | *************************************** ******************** |
| D2^T^ | ACTAGCGAGATGTTGCTACATAATGTCATTTTTTCCCAACAATTCCGACAAAACATAGGT |
| D3 | ACTAGCGAGATGTTGCTACATAATGTCATTTTTTCCCAACAATTCCGACAAAACATAGAT |
| K1 | ACTAGCGAGATGTTGCTACATAATGTCATTTTTTCCCAACAATTCCGACAAAACATAGAT |
| H17 | ACTAGCGAGATGTTGCTACATAATGTCATTTTTTCCCAACAATTCCGACAAAACATAGAT |
|  | ********************************************************** * |
| D2^T^ | GAAATATTTAACGGTTCATTATCTGAAGATCCATCTATTTACGTTTATGTGCCTGCAGTT |
| D3 | GAAATATTTAACGGTTCATTATCTGAAGATCCATCTATTTACGTTTATGTGCCAGCAGTT |
| K1 | GAAATATTTAACGGTTCATTATCTGAAGATCCATCTATTTACGTTTATGTGCCTGCAGTT |
| H17 | GAAATATTTAACGGTTCATTATCTGAAGATCCATCTATTTACGTTTATGTGCCTGCAGTT |
|  | ***************************************************** ****** |
| D2^T^ | GGAGATCGGAGTATGGCACCTGAAGGTCAAACCGGTATCTATGTTTTAATGCCTACGCCA |
| D3 | GGAGATCGGAGTATGGCACCTGAAGGTCAAACCGGTATCTATGTTTTAATGCCTACGCCA |
| K1 | GGAGATCGGAGTATGGCACCTGAAGGTCAAACCGGTATCTATGTTTTAATGCCTACGCCA |
| H17 | GGAGATCGGAGTATGGCACCTGAAGGTCAAACCGGTATCTATGTTTTAATGCCTACGCCA |
|  | ************************************************************ |
| D2^T^ | GAATTAAAAACAGGTCATATTGATTGGAAAGATGAAAATGTGATTAAGAAAGTTAAAGAT |
| D3 | GAATTAAAAACAGGTCATATTAATTGGAAAGATGAAAATGTGATTAGGAAAGTTAAAGAT |
| K1 | GAATTAAAAACAGGTCATATTAATTGGAAAGATGAAAATGTGATTAAGAAAGTTAAAGAT |
| H17 | GAATTAAAAACAGGTCATATTAATTGGAAAGATGAAAATGTGATTAAGAAAGTTAAAGAT |
|  | ********************* ************************ ************* |
| D2^T^ | ATTATCTATCGTCAATTAGAGACAATTGACGTATTAGAGGATGTAAAATCAAATGTTATA |
| D3 | ATTATCTATCGTCAATTAGAGACAATTGACGTATTAGAGGATGTAAAATCAAATGTTATA |
| K1 | ATTATCTATCGTCAATTAGAGACAATTGACGTATTAGAGGATGTAAAATCACATGTTATA |
| H17 | ATTATCTATCGTCAATTAGAGACAATTGACGTATTAGAGGATGTAAAATCACATGTTATA |
|  | *************************************************** ******** |
| D2^T^ | TCTGAAACTGTTTATACACCAATCGATTTTGAAAATGATTATAATGCGAAGTTTGGTACA |
| D3 | TCTGAAACTGTTTATACACCAATCGATTTTGAAAATGATTATAATGCGAAGTTTGGTACA |
| K1 | TCTGAAACTGTTTACACACCAATCGATTTTGAAAATGATTATAATGCGAAGTTTGGTACA |
| H17 | TCTGAAACTGTTTACACACCAATCGATTTTGAAAATGATTATAATGCGAAGTTTGGTACA |
|  | ************** ********************************************* |
| D2^T^ | GCATTTGGTTTAATGCCGACATTAGCCCAAAGTAACTAA--------------------- |
| D3 | GCATTTGGTTTAATGCCGACATTAGCCCAAAGTAACTACTACAGACCTCCAAATGTTTCG |
| K1 | GCATTTGGTTTAATGCCGACATTAGCCCAAAGTAACTACTACAGACCTCCAAATGTTTCG |
| H17 | GCATTTGGTTTAATGCCGACATTAGCCCAAAGTAACTACTACAGACCTCCAAATGTTTCG |
|  | ************************************** |
| D2^T^ | ------------------------------------------------------------ |
| D3 | AGAGATTACAAAGATTTATATTTTGCTGGAGCGAGTACACATCCTTGTGCCGGAGTACCT |
| K1 | AGAGATTACAAAGATTTATATTTTGCTGGAGCGAGTACACATCCTGGTGCCGGAGTACCT |
| H17 | AGAGATTACAAAGATTTATATTTTGCTGGAGCGAGTACACATCCTGGTGCCGGAGTACCT |
|  |  |
| D2^T^ | ------------------------------------------------------------ |
| D3 | ATTGTATTAACTAGCGCTAAAATTACTGTAAATGAAATGTTGAAAGATATAGACAATAAT |
| K1 | ATTGTATTAACTAGCGCTAAAATTACTGTAAATGAAATGTTGAAAGATATAGACAATAAT |
| H17 | ATTGTATTAACTAGCGCTAAAATTACTGTAAATGAAATGTTGAAAGATATAGACAATAAT |
|  |  |
| D2^T^ | ------ |
| D3 | ATTTAG |
| K1 | ATTTAG |
| H17 | ATTTAG |
|  |  |

# Supplementary References

1. De Gelder J, De Gussem K, Vandenabeele P, Moens L. 2007. Reference database of Raman spectra of biological molecules. J Raman Spectrosc 38:1133-1147.

2. Notingher I. 2007. Raman Spectroscopy cell-based Biosensors. Sensors (Basel) 7:1343-1358.

3. Samek O, Telle HH, Harris LG, Bloomfield M, Mack D. 2008. Raman spectroscopy for rapid discrimination of Staphylococcus epidermidis clones related to medical device-associated infections. Laser Phys Lett 5:465-470.

4. Oliveira FSDE, Giana HE, Silveira L. 2012. Discrimination of selected species of pathogenic bacteria using near-infrared Raman spectroscopy and principal components analysis. J Biomed Opt 17.

5. Jehlicka J, Edwards HGM, Orenc A. 2014. Raman Spectroscopy of Microbial Pigments. J Appl Environ Microbiol 80:3286-3295.

6. Czamara K, Majzner K, Pacia MZ, Kochan K, Kaczor A, Baranska M. 2015. Raman spectroscopy of lipids: a review. J Raman Spectrosc 46:4-20.

7. Kumar V, Kampe B, Rosch P, Popp J. 2015. Classification and identification of pigmented cocci bacteria relevant to the soil environment via Raman spectroscopy. Environ Sci Pollut Res Int 22:19317-25.

8. Ayala OD, Wakeman CA, Pence IJ, Gaddy JA, Slaughter JC, Skaar EP, Mahadevan-Jansen A. 2018. Drug-Resistant Staphylococcus aureus Strains Reveal Distinct Biochemical Features with Raman Microspectroscopy. ACS Infect Dis 4:1197-1210.

9. Topfer N, Muller MM, Dahms M, Ramoji A, Popp J, Slevogt H, Neugebauer U. 2019. Raman spectroscopy reveals LPS-induced changes of biomolecular composition in monocytic THP-1 cells in a label-free manner. Integr Biol (Camb) 13:zyz009
